# POU1F1 induces cancer stem cell-*like* traits in breast cancer cells by IL-6/JAK2/STAT3 activation and enrichment of ALDH

**DOI:** 10.1038/s41523-026-00929-w

**Published:** 2026-03-19

**Authors:** Leandro Avila, Samuel Seoane, Sandra Rodriguez-Gonzalez, Magda Gois, Mª Efigenia Arias, David Martinez-Delgado, Noemi Gomez-Lado, Tomas Garcia-Caballero, Pablo Aguiar, Roman Perez-Fernandez

**Affiliations:** 1 Department of Physiology & Center for Molecular Medicine and Chronic Diseases (CIMUS), University of Santiago de Compostela, Santiago de Compostela, Spain; 2 Department of Obstetrics and Gynecology, Health Research Institute of Santiago de Compostela (IDIS), University of Santiago de Compostela, Santiago de Compostela, Spain; 3 Stem Cell and Human Disease, Center for Molecular Medicine and Chronic Diseases (CIMUS), University of Santiago de Compostela, Santiago de Compostela, Spain; 4 Molecular Imaging and Theragnosis Group, Center for Molecular Medicine and Chronic Diseases (CIMUS) and Research Institute of Santiago de Compostela (IDIS), University of Santiago de Compostela, Santiago de Compostela, Spain; 5 Department of Morphological Sciences, Health Research Institute of Santiago de Compostela (IDIS), University of Santiago de Compostela, Santiago de Compostela, Spain

**Keywords:** Cancer, Cell biology, Oncology

## Abstract

Breast cancer stem cells (BCSCs) have been proposed as the cause of resistance to conventional treatments and of breast cancer recurrence and metastasis. This study provides compelling evidence for the role of the transcription factor POU1F1 in the increase of BCSC-*like*. Using POU1F1-overexpressing and knock-down breast cancer cell lines, as well as immunodeficient mouse models, our data demonstrate that POU1F1 induces a BCSC-*like* phenotype in breast tumor cells by deregulating markers such as CD24, CD44, CD133, and ALDH. These phenotypic modifications correlate with functional changes, i.e., increased clonogenicity, mammosphere formation, and glycolysis. In addition, we found that a subpopulation of MCF-7 cells with overexpression of POU1F1 and elevated ALDH expression exhibits both a high tumor-initiating capacity and increased resistance to chemotherapy and radiotherapy treatments. Mechanistically, these features are mediated by POU1F1 activation of the IL-6/JAK2/STAT3 pathway and up-regulation of ALDH. Janus kinase inhibitors and monoclonal anti-IL6 receptor antibodies significantly decrease ALDH expression, colony, and mammosphere formation, suggesting possible use of pharmacological inhibitors of the IL-6/JAK2/STAT3 pathway in breast tumors with elevated POU1F1 levels.

## Introduction

During cancer progression, cancer cells can acquire molecular and phenotypic changes, an ability known as cellular plasticity^1^. Cell plasticity, also known as lineage plasticity, could be defined as the ability of a cell to reprogram and change its phenotype identity^1–3^. The acquisition of epithelial-mesenchymal transition (EMT)^4–6^ and cancer stem cell-*like* (CSC-*like*) states are two of the best-known cases of tumor cell plasticity, and both states have been linked^7,8^. CSCs, in particular breast cancer stem cells (BCSCs), have been proposed as the cause of therapy resistance, recurrence, and metastasis of breast cancer^9^. Thus, metastasis could be initiated by a subpopulation of cancer cells (also called “tumor-initiating cells”) that possess stem cell-*like* properties, being more favorable to reinitiate tumor growth at distant sites and to acquire resistance to therapy, resulting in increased aggressiveness and relapse^10–13^. The most common markers for identifying BCSCs are CD24^−/low^, CD44^+/high^, CD133^+^, and ALDH^+^, which are also found in several other types of tumors^14^. In addition, BCSCs have common functional characteristics, such as higher clonogenicity, formation of three-dimensional (3D) structures in cultures (called spheroids or mammospheres), and tumor-initiating capacity in vivo^15,16^.

The POU class 1 homeobox 1 (POU1F1 or Pit1) transcription factor belongs to the Pit1-Oct3/4-Unc86 (POU) family of transcription factors that play key roles in cell proliferation, cell lineages, and regulation of terminal differentiation^17^. POU1F1 was first discovered in the pituitary gland, being responsible for cell differentiation and as a transcriptional activator during its organogenesis^18–20^. However, POU1F1 is also expressed in other tissues, such as the mammary gland. In breast cancer, POU1F1 expression is higher than in normal cells and induces cell proliferation^21,22^. In addition, POU1F1 upregulates Snai1 and induces EMT, promoting tumor growth and metastasis^23^. POU1F1 also influences the tumor microenvironment (TME), modifying the phenotype of fibroblasts and recruiting and differentiating normal macrophages into tumor-associated macrophages (TAMs) to promote cancer progression^24–26^.

Given that POU1F1 is related to EMT-cell plasticity and metabolism modification, we intend to analyze the relationship between POU1F1 and BCSCs. Using BC cell lines and immunodeficient mouse models, we evaluated changes in BCSC markers, colony growth, mammosphere formation, tumor initiation capacity, and resistance to hormone and radiotherapy after overexpression and knockdown of POU1F1. Additionally, we investigated the mechanisms by which these changes may occur.

Our results indicate that POU1F1 enhances stem-like features in BC cells and suggest a therapeutic strategy for treating POU1F1-overexpressing breast tumors.

## Results

### POU1F1 induces stem-like features in MCF-7 cells

**Fig. 1:**
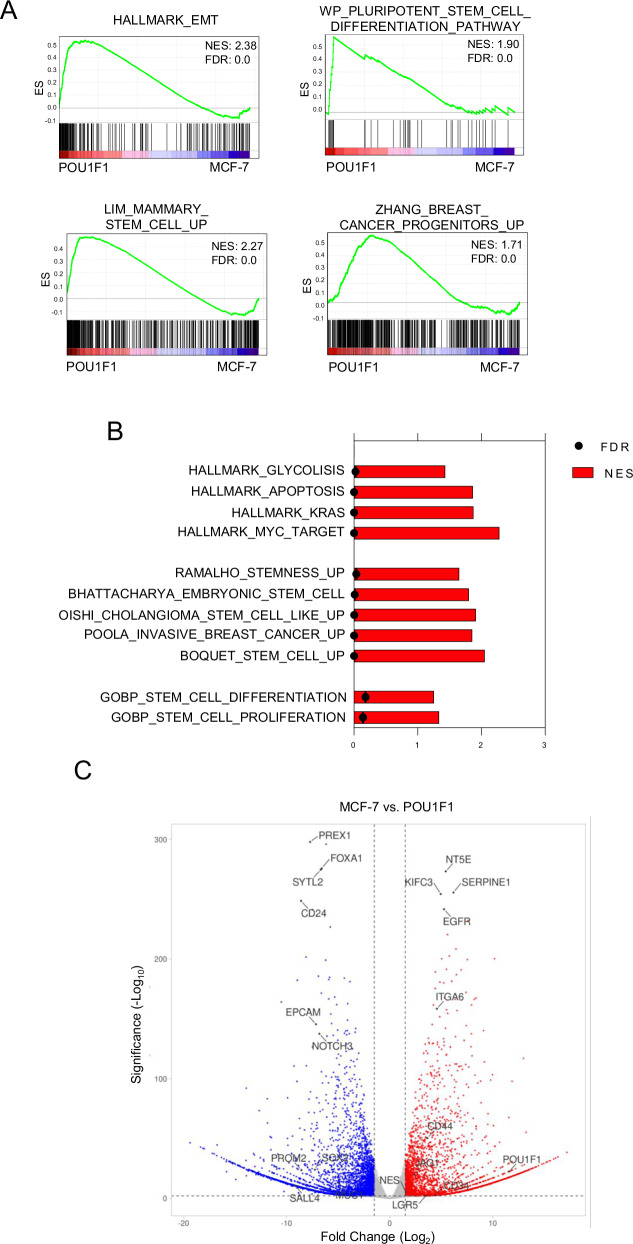
RNA-seq data of MCF-7 cells without/with POU1F1 overexpression. **A** GSEA plot of enrichment in EMT, pluripotent stem cell differentiation pathway, mammary stem cell, and breast cancer progenitors geneset. **B** Dataset enrichment graph of signature associated with stem cell data by RNA-seq from POU1F1-overexpressing MCF7 cells (POU1F1) vs control MCF7 cells (transfected with the pLV-puro-EF1A empty vector) (GSE287812). **C** Volcano plot shows significant downregulated (blue dots) and upregulated (red dots) CSC markers in MCF-7 versus POU1F1 cells.

To investigate whether POU1F1 causes stem cell-related modifications in tumor cells, the luminal A subtype of human breast cancer MCF-7 cells was stably transfected with the POU1F1 overexpression vector (Supplementary Fig. 1A, B), and a gene set enrichment analysis (GSEA) was performed. We compared the transcriptomes of MCF-7 (which exhibit low endogenous POU1F1 expression) with those of MCF-7 cells overexpressing POU1F1 (hereinafter referred to as POU1F1 cells). This analysis revealed significant enrichment of gene signatures associated with cellular plasticity and stem cell activation in the POU1F1 cells, suggesting that POU1F1 promotes transcriptional programs linked to cancer stem cell-like features (Fig. 1A, B). The volcano plot analysis of RNA-seq data in POU1F1 cells reveals deregulation of gene expression related to cancer stem cell differentiation, e.g., CD44, ITGA6 (CD49f), EGFR, NT5E, CD24, EpCAM, FOXA1, and NOTCH3 (Fig. 1C). These data were further compared with data obtained from an RNA-seq in human mammary epithelial cells (HMEC) with and without POU1F1 overexpression. Similar changes were not observed in the aforementioned genes related to cancer stem cell differentiation (Supplementary Fig. 2A–C).

**Fig. 2:**
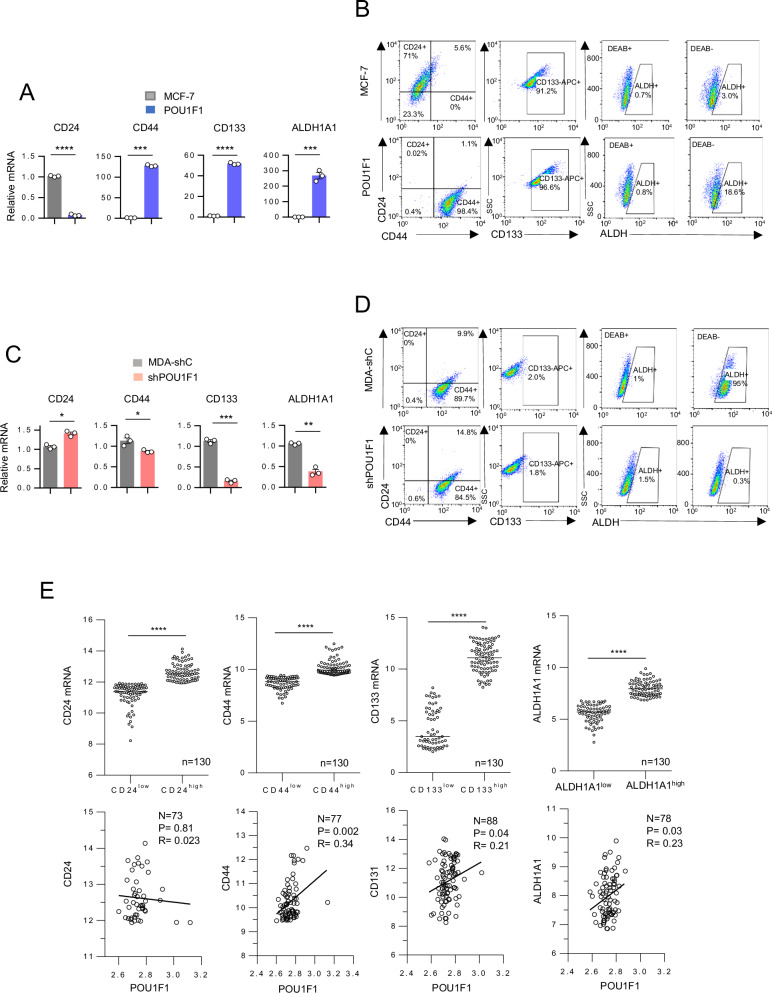
POU1F1 induces a BCSC-*like* phenotype from breast cancer cells. **A** qPCR of CD24, CD44, CD133, and ALDH1A1 mRNA in MCF-7 cells transfected with the empty vector (MCF-7), and with the POU1F1 overexpression vector (POU1F1). **B** Flow cytometry analysis of CD24/CD44, CD133, and ALDH in MCF-7 and POU1F1 cells (DEAB was used to establish baseline fluorescence to define the ALDEFLUOR positive area). **C** qPCR of CD24, CD44, CD133, and ALDH1A1 mRNA in control MDA-MB-231 cells (MDA-shC) and MDA-MB-231 cells with POU1F1 knock-down (shPOU1F1). **D** Flow cytometry analysis of CD24/CD44, CD133, and ALDH in MDA-shC and shPOU1F1 cells, as described in (**B**). **E** Dispersion plot of CD24, CD44, CD133, and ALDH1A1 mRNA levels in human breast tumors (*n* = 130) (GSE65194). CD24, CD44, CD133, and ALDH1A1 were classified according to their mRNA expression levels: high (above the mean) or low (below the mean). Correlation analysis of mRNA expression. Results are represented as mean ± SEM. ∗*p* < 0.05, ∗∗*p* < 0.01, ∗∗∗*p* < 0.001, ∗∗∗∗p < 0.0001.

Subsequently, we selected well-known BCSC markers, such as CD24, CD44, CD133, and ALDH, and carried out real-time PCR in MCF-7 control cells as well as in POU1F1 cells. We found a significant increase in CD44 (*P* < 0.001), CD133 (*P* < 0.0001), and ALDH1A1 (*P* < 0.01) mRNA and a decrease (*P* < 0.0001) in the CD24 marker in POU1F1 cells (Fig. 2A). A flow cytometry was also carried out in these cells to evaluate protein expression. Double-labeling indicates a strong shift from CD24^+^/CD44^-^ markers in MCF-7 control cells to CD24^-^/CD44^+^ in POU1F1 cells. In addition, there was also an increased protein expression in CD133 and ALDH activity in POU1F1 cells (Fig. 2B). Conversely, the MDA-MB-231 cell line (triple-negative subtype, with higher endogenous levels of POU1F1 than MCF-7 cells) after POU1F1 knockdown (Supplementary Fig. 3A, B) presents a significant change in the BCSC-marker profile, i.e., higher CD44, CD133, and ALDH1A1 mRNA expression in control MDA-MB-231 cells (MDA-shC), as compared to POU1F1-knocked-down MDA-MB-231 cells (shPOU1F1) (Fig. 2C). Protein levels of CD44 and CD133 also decreased in shPOU1F1 cells, with a striking decrease in ALDH activity from 95% in control cells to 0.33% (Fig. 2D). Next, we correlated POU1F1 mRNA expression and BCSC markers in the GSE65194 database of human breast tumor samples. Tumors were separated according to high (above the mean) or low (below the mean) CD24, CD44, CD133, and ALDH1A1 mRNA expression (*n* = 130 tumors, Fig. 2E, upper panel). As shown in Fig. 2E, lower panel, we found a correlation between POU1F1 and CD44 (R = 0.34, P = 0.002), CD133 (R = 0.21, *P* = 0.04), and ALDH1A1 (R = 0.23, *P* = 0.03) mRNA expression.

### A subpopulation of POU1F1 cells with high ALDH expression (POU1F1-ALDH^high^) has strong BCSC-like characteristics

**Fig. 3:**
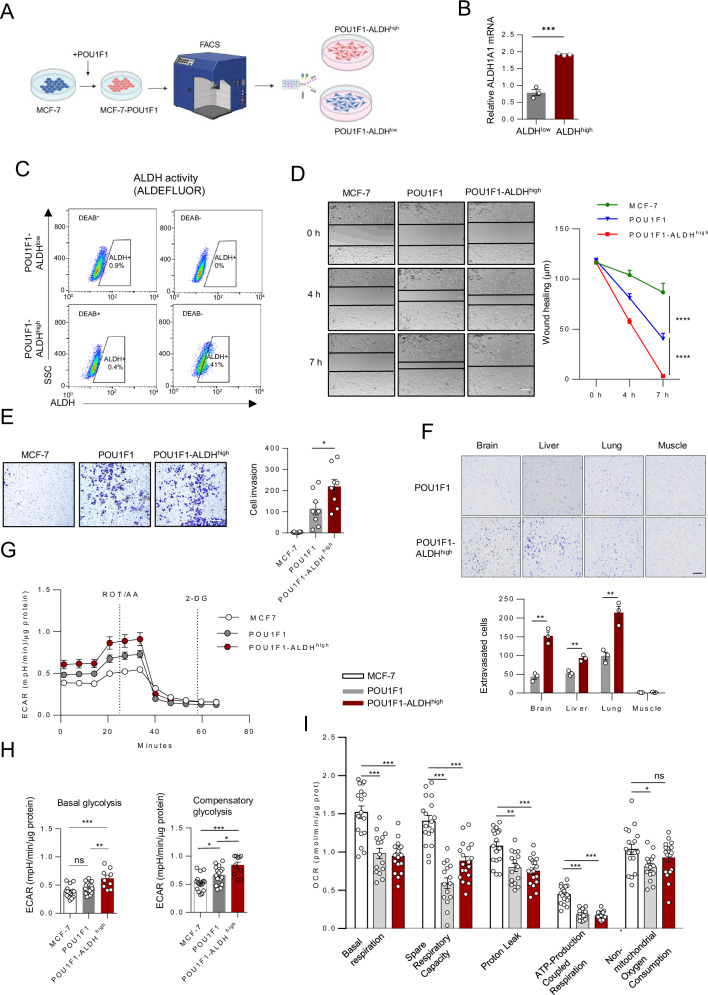
The POU1F1-ALDH^high^ subpopulation has strong BCSC-*like* characteristics. **A** Schematic of isolating the POU1F1-ALDH^high^ subpopulation. **B** qPCR of ALDH1A1 mRNA in ALDH^low^ and ALDH^high^ cells after sorting MCF-7 cells with POU1F1 overexpression. **C** Flow cytometry analysis of ALDH activity (ALDEFLUOR assay) in POU1F1-ALDH^low^ and POU1F1-ALDH^high^ cells. **D** Wound-healing assay of control MCF-7 cells (transfected with the empty vector), MCF-7 cells with POU1F1 overexpression (POU1F1), and MCF-7 cells with POU1F1 overexpression and ALDH^high^ (POU1F1-ALDH^high^) at 0, 4, and 7 h (scale bar: 100 μm). **E** Transwell invasion assay and quantitative analysis in control MCF-7, POU1F1, and POU1F1-ALDH^high^ cells after 24 h (scale bar: 100 μm). **F** Transwell invasion assay and quantitative analysis in control MCF-7, POU1F1, and POU1F1-ALDH^high^ cells. Cells are plated into the upper chamber, and the lower chamber is filled with mouse tissue extract (brain, liver, lung, and muscle used as a negative control). **G** Representative ECAR Glycolytic Rate Assay profile in MCF7, POU1F1, and POU1F1-ALDH^high^ cells. **H** Quantification of basal glycolysis and compensatory glycolysis. **I** Quantification of mitochondrial respiration parameters. Results are represented as mean ± SEM. ∗*p* < 0.05, ∗∗*p* < 0.01, ∗∗∗*p* < 0.001.

To delve into how POU1F1-overexpressing MCF-7 cells modulate their phenotype, flow cytometry was performed to separate two populations of cells according to ALDH1A1 expression: (a) POU1F1-ALDH^low^, and (b) POU1F1-ALDH^high^ (Fig. 3A). As shown in Fig. 3B, C, cells enriched with ALDH1A1 mRNA and protein (POU1F1-ALDH^high^) were obtained. Wound healing and invasion assays were performed to analyze the functional properties of these cells, particularly their migratory and invasive capacity. A significant increase in both migration (*P* < 0.0001) and invasion (*P* < 0.05) was observed in POU1F1-ALDH^high^ cells as compared to POU1F1 cells (Fig. 3D, E). POU1F1-ALDH^high^ cells metastasize significantly more in lung, brain, and liver tissues than POU1F1 cells (Fig. 3F). As previously shown^25^, POU1F1 cells have a glycolytic profile because POU1F1 transcriptionally upregulates the key lactate dehydrogenase A (LDHA) enzyme, which leads MCF-7 cell metabolism from pyruvate to lactate production. Next, the glycolytic activity of the POU1F1-ALDH^high^ subpopulation was assayed by determining extracellular acidification rate (ECAR), basal glycolysis, and compensatory glycolysis. Figure 3G, H shows that POU1F1-ALDH^high^ cells significantly increase both basal (*P* < 0.01) and compensatory (*P* < 0.05) glycolysis compared with POU1F1 cells. Mitochondrial respiration was also evaluated. POU1F1 cells showed a significant reduction in basal respiration (*P* < 0.001), spare respiratory capacity (*P* < 0.001), proton leak (*P* < 0.05–0.01), and ATP-linked respiration (*P* < 0.001) compared to MCF 7 cells, indicating OXPHOS decrease. The POU1F1-ALDH^high^ cells maintained a low OXPHOS profile, but displayed no significant decrease in non-mitochondrial oxygen consumption vs MCF-7 cells (Fig. 3I).

### Colony formation and mammosphere density are increased in POU1F1-ALDH^high^ cells

**Fig. 4:**
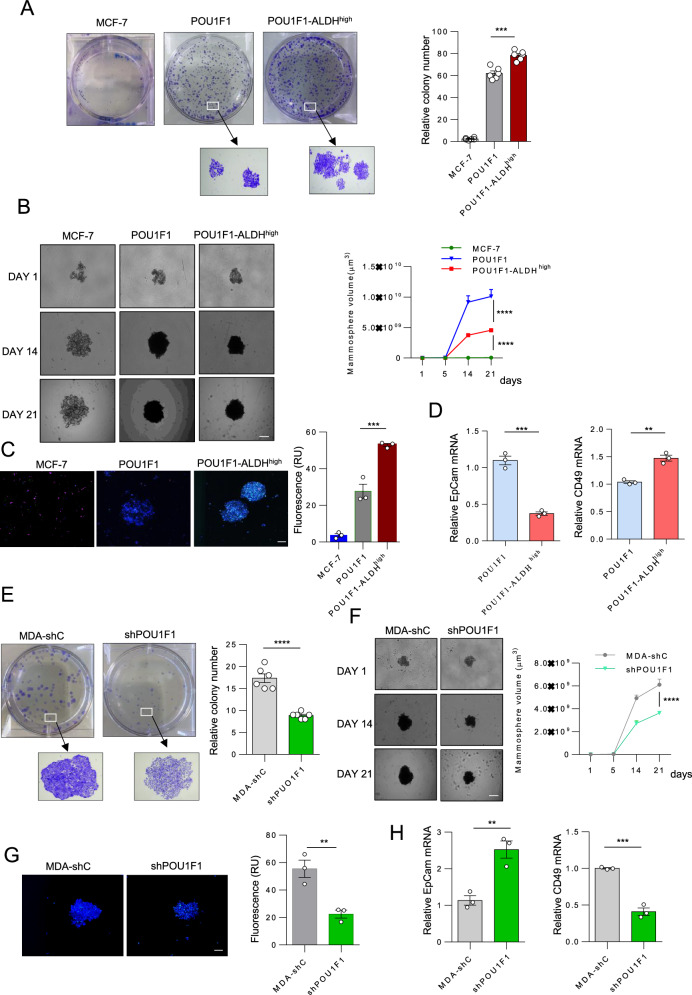
POU1F1-ALDH^high^ cells display BCSC-*like* features. **A** Colony formation and quantitative analysis of control MCF-7, POU1F1, and POU1F1-ALDHhigh cells. **B** Mammosphere formation and volume in MCF-7, POU1F1, and POU1F1-ALDH^high^ cells at 1, 14, and 21 days (scale bar: 200 μm). **C** Confocal microscopy and quantification of fluorescence (live cells) in mammospheres (scale bar: 200 μm). **D** qPCR of EpCAM and CD49 mRNA in POU1F1 and POU1F1-ALDH^high^ cells. **E** Colony formation and quantitative analysis in MDA-MB-231 control cells (MDA-shC) and MDA-MB-231 cells with knockdown of POU1F1 (shPOU1F1). **F** Mammosphere formation and volume in MDA-shC and shPOU1F1 at 1, 14, and 21 days. **G** Confocal microscopy at the midsection of mammospheres in control MDA-shC and shPOU1F1 cells. **H** qPCR of EpCAM and CD49 mRNA in control MDA-shC and shPOU1F1 cells. Results are shown as mean ± SEM. ∗∗*p* < 0.01, ∗∗∗*p* < 0.001.

BCSCs are characterized by an increased size and number of forming colonies and the ability to form mammospheres. Thus, we studied these parameters in MCF-7, POU1F1, and POU1F1-ALDH^high^ cells. As shown in Fig. 4A, high levels of ALDH significantly increase colony number (*P* < 0.001), even though mammosphere volume is larger in POU1F1 cells than in POU1F1-ALDH^high^ cells (*P* < 0.0001) (Fig. 4B). In addition, confocal microscopy indicated that the mammospheres from POU1F1-ALDH^high^ cells have a significantly (*P* < 0.001) higher density compared to POU1F1 cells, as measured by the number of live cells (Fig. 4C). No significant differences were found in apoptosis (caspase 3/7) or cell death (PI) between MCF-7 and POU1F1 cells or between POU1F1 and POU1F1-ALDH cells (Supplementary Fig. 4A). The CD49 and EpCAM markers, characteristic of progenitor luminal cells (CD49^+^) and differentiated luminal cells (EpCAM^+^), were evaluated by qPCR in these mammospheres, showing that POU1F1-ALDH^high^ cells present significantly low EpCAM (*P* < 0.001) and high CD49 (*P* < 0.01) mRNA expression as compared to POU1F1 cells (Fig. 4D). Conversely, POU1F1 knockdown in MDA-MB-231 (shPOU1F1) cells indicated that cells with low levels of POU1F1 have a significantly lower capacity to form colonies (*P* < 0.0001, Fig. 4E). Moreover, mammospheres presented lower volume (*P* < 0.0001, Fig. 4F), lower density (*P* < 0.01, Fig. 4G), reduced, but no significant, cell death and apoptosis (Supplementary Fig. 4B), and presented significantly high EpCAM (*P* < 0.01) and low CD49 (*P* < 0.001) mRNA expression as compared to MDA-shC cells (Fig. 4H).

### POU1F1-ALDH^high^ cells have a greater capacity to initiate tumors and greater resistance to treatment

**Fig. 5:**
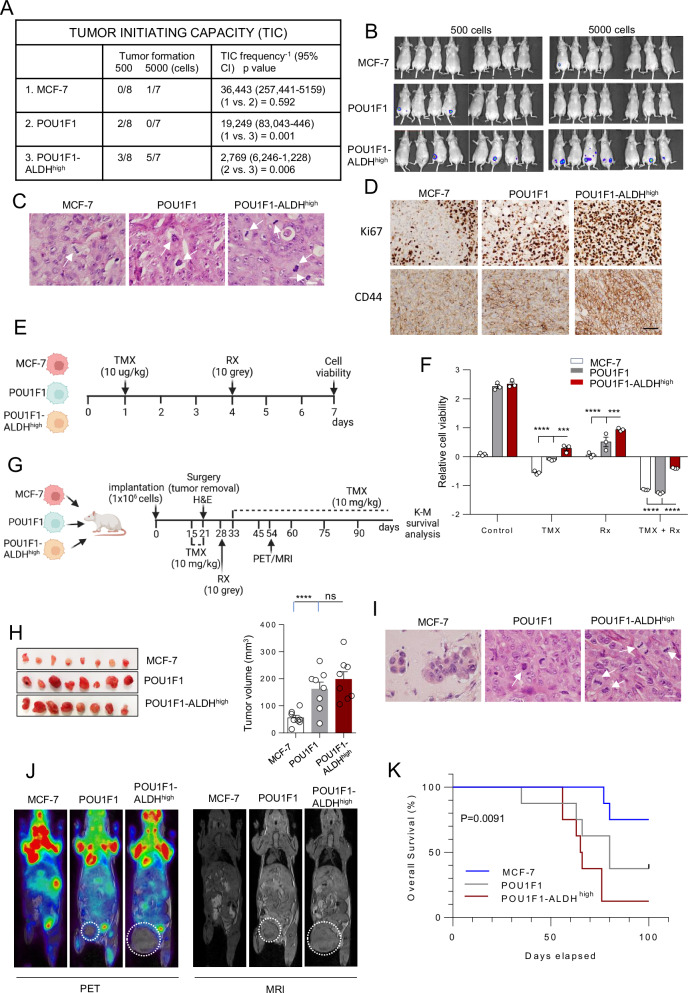
Evaluation of tumor-initiating capacity (TIC) and resistance to treatment. **A** TIC of control MCF-7 cells (transfected with the empty vector), POU1F1-overexpressing MCF-7 cells (POU1F1), and POU1F1-overexpressing cells with elevated ALDH levels (POU1F1-ALDH^high^) implanted into the fourth mammary fat pad of mice (5 × 10^2^ cells, *n* = 8, and 5 × 10^3^ cells, *n* = 7). Tumors were monitored for 6 weeks to assess tumorigenicity. **B** Luminescence of tumors (IVIS images) was recorded at 6 weeks. **C** Example of H&E staining in tumors. White arrows indicate cell mitosis (scale bar: 300 μm). **D** Immunohistochemistry of Ki67 (low proliferation index in MCF7 cells, intermediate in POU1F1, and high in POU1F1-ALDH^high^ cells) and CD44 (1+ in MCF7, 2+ in POU1F1, and 3+ in POU1F1-ALDH^high^ tumors) (scale bar: 200 μm). **E** Experimental design to evaluate hormone- and radiotherapy resistance in MCF-7, POU1F1, and POU1F1-ALDH^high^ cells (TMX: tamoxifen; RX: radiotherapy). **F** Relative cell viability in MCF-7, POU1F1, and POU1F1-ALDH^high^ cells after hormone therapy, radiotherapy, and both. **G** Experimental design to evaluate hormone- and radiotherapy resistance in vivo after xenografting of MCF-7, POU1F1, and POU1F1-ALDH^high^ cells in mice (*n* = 8 per group). **H** Tumors were excised 21 days post-implantation of cells, and tumor volume was calculated. **I** Examples of H&E staining in tumors. White arrows indicate cell mitosis (scale bar: 300 μm). **J** [^18^F] FDG PET/MRI assessed glucose uptake and tumor growth on day 54 in two mice per group. The dotted circle highlights the tumor. **K** Kaplan–Meier overall survival curves. Results are presented as mean ± SEM. ∗∗∗*p* < 0.001, ∗∗∗∗*p* < 0.0001, ns: not significant.

We next evaluated the effects of POU1F1-ALDH^high^ cells on tumor developing capacity. Immunodeficient BALB/c-nu female mice were injected in the mammary fat pad with either 500 or 5000 cells of MCF-7, POU1F1, and POU1F1-ALDH^high^, and a tumor-initiating capacity (TIC) assay was performed. Of the mice injected with POU1F1-ALDH^high^ cells, 3 out of the 8 mice injected with 500 cells and 5 out of the 7 mice injected with 5000 cells developed tumors after 6 weeks, compared with 2 out of 8 mice and 0 out of 7 mice in the POU1F1-injected group. TIC frequency was significantly higher (2769) in POU1F1-ALDH^high^ compared to POU1F1 (19,249) (*P* = 0.006) and MCF-7 (36,443) (*P* = 0.001) injected mice (Fig. 5A). Figure 5B, C shows tumor luminescence, and examples of H&E staining. The mitotic count is clearly increased in POU1F1 (61 mitoses/10 HPF) and POU1F1-ALDH^high^ cells (199 mitoses/10 HPF) compared to MCF-7 control cells (18 mitoses/10 HPF). Higher Ki67 positivity in POU1F1-ALDH^high^ tumors (87.4%) compared to POU1F1 (67.3%) and MCF-7 (45.2%) tumors was also observed (Fig. 5D). CD44 also showed clearly higher expression in POU1F1 (score of 2 +) and POU1F1-ALDH^high^ (3 +) with respect to MCF-7 cells (1 +) (Fig. 5D). As shown in Supplementary Fig. 5, tumors from MCF-7 injected cells are smaller than those injected with POU1F1 cells.

We developed an in vitro study using hormone therapy and radiotherapy to evaluate tumor cell resistance to treatment, as is routinely done in patients for the treatment of Luminal A subtype breast tumors. Tamoxifen (TMX), a selective estrogen receptor modulator, clinically used for the treatment of hormone receptor-positive Luminal A subtype of breast tumors (such as the MCF-7 cell line), was administered to MCF-7, POU1F1, and POU1F1-ALDH^high^ cells, and 72 h later, a radiation dose was given. Cell viability was then analyzed (Fig. 5E). Higher cell viability, i.e., higher resistance to treatment (TMX, radiotherapy, and TMX + radiotherapy) was observed in POU1F1-ALDH^high^ cells compared to the other groups (Fig. 5F). We also tested the resistance to treatment in vivo (Fig. 5G). After treatment with TMX on day 15 for 6 days, the tumor was surgically excised under anesthesia. Figure 5H shows no significant difference in tumor volume between mice injected with POU1F1-ALDH^high^ cells and those injected with POU1F1 cells. Tumor H&E staining indicates a high number of cell mitoses in the POU1F1-ALDH^high^ group (62 mitoses/10 HPF) as compared to the POU1F1 (41 mitoses/10 HPF) and MCF-7 (3 mitoses/10 HPF) groups (Fig. 5I). After a single dose of radiotherapy on the excised tumor area on day 28, mice were treated again with TMX from day 33 until death, and overall survival was assessed by Kaplan–Meier analysis. In addition, on day 54, metabolic tumor activity (glucose uptake) was analyzed in two mice per group using [^18^F] FDG PET/MRI (Fig. 5J). Control MCF-7 mice showed no evidence of tumors in either the MRI or PET images. POU1F1 mice showed an active tumor in one mouse, which was small in the morphological images (indicated by a dotted circle) but with significant metabolic activity at the tumor edges. POU1F1-ALDH^high^ mice showed large, spherical tumors in the MRI images, with metabolic activity primarily located at the tumor edges, while the central region of the tumor appears inactive, likely indicating necrotic tissue (Fig. 5J and Supplementary Fig. 6). Finally, the Kaplan–Meier survival analysis shows a clear difference in overall survival among the three groups over time (*P* = 0.009) (Fig. 5K). MCF-7 had the highest survival rate, with no significant decline until later time points. POU1F1-ALDH^high^ demonstrated the poorest survival, with a rapid and steep decline in survival rates observed early in the study period compared with POU1F1 cells.

To explore the potential relationship between POU1F1/ALDH1A1 expression and clinical outcome, POU1F1 and ALDH mRNAs were analyzed in a dataset of human breast cancer patients. Elevated POU1F1 and ALDH expression were associated with significantly worse outcomes in patients. Kaplan–Meier analysis showed that the high-expression group had reduced recurrence-free survival (RFS) (*P* = 7.1 × 10^−^⁸), reduced overall survival (OS) (*P* = 0.00012), and reduced distant metastasis-free survival (DMFS) (P = 0.00062) compared to the low-expression group. These data indicate that high expression of both genes defines a population with a worse clinical prognosis (Supplementary Fig. 7).

### POU1F1 activates the IL-6/JAK2/STAT3 pathway

**Fig. 6:**
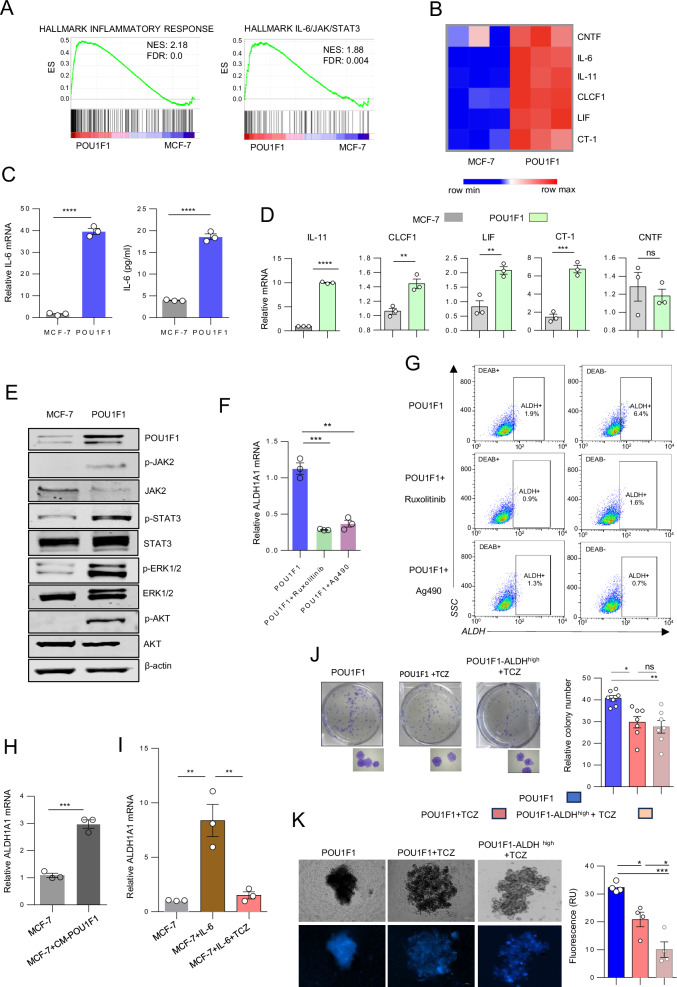
POU1F1 regulates the IL-6/JAK2/STAT3 pathway. **A** GSEA plot of enrichment in inflammatory response and IL-6/JAK2/STAT3 pathway of the RNA-seq data from POU1F1-overexpressing MCF-7 (POU1F1) cells vs. control MCF-7 cells (transfected with the empty vector). **B** Heatmap of members of IL-6 family of interleukins with and without POU1F1 overexpression in MCF-7 cells. **C** qPCR of IL-6 mRNA and ELISA of IL-6 protein in culture medium after 72 h. **D** qPCR of IL-11, CLCF1, LIF, CT-1, and CNTF mRNA expression in MCF-7 and POU1F1 cells. **E** Immunoblots of POU1F1, phosphorylated (p) JAK2 (p-JAK2), JAK2, p-STAT3, STAT3, p-ERK1/2, ERK1/2, p-AKT, AKT, β-actin in control MCF-7 and POU1F1 cells. **F** qPCR of ALDH1A1 mRNA in POU1F1 cells before and after treatment with Ruxolitinib and Ag490. **G** Flow cytometry analysis of ALDH activity (ALDEFLUOR assay) in POU1F1 cells before and after treatment with Ruxolitinib and Ag490. **H** qPCR of ALDH1A1 mRNA expression in untreated MCF-7 cells and after treatment with conditioned medium (CM) from POU1F1-overexpressing MCF-7 cells. **I** qPCR of ALDH1A1 mRNA in MCF-7 control cells, after treatment with IL-6, and treated with IL-6+Tocilizumab (TCZ, anti-IL-6 receptor antibody). **J** Colony formation in POU1F1 and POU1F1-ALDH^high^ cells after treatment with TCZ. **K** Mammosphere images and density in POU1F1 and POU1F1-ALDH^high^ cells treated with TCZ. Live cells were detected by fluorescence at the midsection of mammospheres (scale bar: 200 μm). Results are presented as mean ± SEM. ∗∗*p* < 0.01, ∗∗∗*p* < 0.001, ∗∗∗∗*p* < 0.0001.

To elucidate the possible mechanisms involved in the phenotype change of breast cancer cells to BCSC-*like* mediated by POU1F1, bioinformatic analyses for inflammatory and cytokine processes involved in CSC activity were carried out in an RNA-seq of MCF-7 and POU1F1 cells. POU1F1 overexpression induced a clear enrichment of hallmarks of inflammatory response and Interleukin-6/Janus kinase 2/signal transducer and activator of transcription 3 (IL-6/JAK2/STAT3) signaling pathway signatures (NES = 2.18, FDR = 0.00, and NES = 1.88, FDR = 0.004, respectively) (Fig. 6A). The IL-6/JAK2/STAT3 pathway has been involved in CSC marker regulation in several tumor types^27–29^. In addition to IL-6, other members of the IL-6 cytokine family include IL-11, oncostatin M (OSM), leukemia inhibitory factor (LIF), cardiotrophin 1 (CT-1), ciliary neurotrophic factor (CNTF), cardiotrophin-like cytokine factor 1 (CLCF1), IL-27, IL-35, and IL-39^30^. A heatmap from our RNA-seq data indicates a significant up-regulation in CNTF, IL-6, IL-11, CLCF1, LIF, and CT-1 following POU1F1 overexpression (Fig. 6B). Indeed, Volcano plot analysis revealed that multiple cytokines were upregulated after POU1F1 overexpression, including IL-6 and IL-11 with a fold change >3.5 (Supplementary Fig. 8A). Both qPCR and ELISA analyses confirm that POU1F1 significant (*P* < 0.0001) increases IL-6 mRNA expression, and IL-6 protein in culture medium (Fig. 6C). Also, IL-11, CLCF1, LIF, and CT-1 mRNA significantly increased after POU1F1 although not CNTF mRNA (Fig. 6D). Indeed, POU1F1 induces phosphorylation of proteins involved in the JAK2/STAT3 signaling pathway, such as JAK2, STAT3, ERK1/2, and AKT (Fig. 6E). To study whether stimulation of the IL-6/JAK2/STAT3 pathway by POU1F1 might in turn regulate ALDH, POU1F1 cells were treated with two Janus Kinase inhibitors (JKI), Ruxolitinib and AG490 (Supplementary Fig. 8B), and ALDH1A1 expression was evaluated. A significant decrease in ALDH1A1 mRNA and protein activity was found (Fig. 6F, G). To analyze whether the upregulation of ALDH1A1 by POU1F1 was produced through the IL-6/JAK2/STAT3 pathway, experiments were performed to activate or inhibit this pathway. MCF7 cells were treated with (a) conditioned medium (CM) from POU1F1 cells (CM-POU1F1) (Fig. 6H), (b) IL-6 ligand (IL-6) (Fig. 6I), and (c) an anti-IL-6 receptor monoclonal antibody (TCZ, used clinically to block the IL-6 pathway) (Fig. 6I). The results demonstrate a significant increase in ALDH1A1 mRNA expression after administration of both CM-POU1F1 (*P* < 0.001) and IL-6 (*P* < 0.01), as well as a significant decrease (*P* < 0.01) after administration of TCZ. To confirm the functional effects of IL-6/JAK2/STAT3 pathway blockade, colony and mammosphere formation were analyzed after TCZ administration to POU1F1 and POU1F1-ALDH^high^ cells. Treatment with TCZ significantly reduced colony formation (Fig. 6J) and the density of the mammospheres as compared to control spheroids (Fig. 6K).

## Discussion

POU1F1 is a well-known transcription factor involved in embryonic and adult pituitary development and has also been analyzed for its role in cancer^23,26^. This study provides compelling evidence for the role of POU1F1 in increasing cancer stem cell-*like* features in breast cancer cells. Our data demonstrate that POU1F1 induces a BCSC-*like* phenotype in breast tumor cells by deregulating markers such as CD24, CD44, CD133, and ALDH. These phenotypic modifications correlate with functional changes, i.e., higher clonogenicity, increased mammosphere formation, and increased glycolytic metabolism. In addition, using immunodeficient mouse models, we found that a subpopulation of ALDH^high^ cells obtained from POU1F1-overexpressing MCF-7 cells has high tumor-initiating capacity, high resistance to hormone and radiotherapy, and reduced overall survival. Mechanistically, these actions are mediated by POU1F1 through activation of the IL-6/JAK2/STAT3 pathway.

The overexpression of POU1F1 in the luminal A subtype of the breast cancer MCF-7 cell line (with low POU1F1 endogenous levels) induces a phenotypic shift from CD24⁺/CD44⁻ and ALDH^low^ into CD24⁻/CD44⁺ and ALDH^high^, characteristics of BCSCs, as first described by Al-Haji et al.^15^. Conversely, in the triple-negative MDA-MB-231 cell line (with higher endogenous POU1F1 levels), blockade of POU1F1 results in decreased expression of CD44, CD133, and a striking drop in ALDH activity. This shift in BCSC markers correlated with POU1F1 mRNA expression levels in human breast tumor databases, independently of the tumor subtype. However, POU1F1 overexpression does not induce phenotypic changes in normal cells (HUMEC), suggesting that the effects of POU1F1 are restricted to the specific tumor context.

The subpopulation isolated from POU1F1 cells, identified as POU1F1-ALDH^high^ cells, exhibits the highest functional capacities, including enhanced migration and invasion compared to other subpopulations. These findings highlight its distinctive potential and are consistent with previously described characteristics of cells with similar phenotypes^31,32^.

POU1F1 has been shown to enhance glycolysis, facilitating stem cell-*like* properties in gastric carcinoma cells^33^. Our results agree, also showing that POU1F1 represses OXPHOS and drives glycolytic metabolism, which may correspond to the high energy demands of BCSCs during growth and proliferation^34^. POU1F1-ALDH^high^ cells show a marked increase in compensatory glycolysis, indicating metabolic flexibility and adaptability, for example, to hypoxia or oxidative stress^35^. In fact, it has recently been shown that ALDH^high^ BCSCs reduce reactive oxygen species, leading to BCSC expansion and tumor initiation in mice^36^.

In addition to the higher cell density observed in POU1F1-ALDH^high^ mammospheres, the CD49f^+^/EpCAM^−^ phenotype acquired by these cells could be associated with the ability to generate different structures within the mammary gland, including basal, luminal progenitor, and mature cell types^37^, suggesting a potential role in differentiation and organization of the tumor microenvironment (TME). Moreover, this phenotype has been related to a high metastatic potential in tumor cells and an increased risk of recurrence^38^. Our data also indicate that the POU1F1-ALDH^high^ subpopulation exhibits greater tumor-initiation capacity and greater resistance to conventional therapies, a process that may be related to elevated ALDH levels. Furthermore, we have found a significant relationship between high expression of POU1F1 / ALDH1A1 and a poor clinical outcome in breast cancer patients. Tumor-initiating cells create a microenvironment that spatially favors tumor progression and drug resistance^39,40^. Specifically in breast cancer, ALDH1A1 was described as promoting tumor progression through immune system modulation^41^, and downregulation of ALDH1A1 increases the sensitivity of tumor cells to treatments^42^. However, POU1F1 overexpression alone is sufficient to induce a broad cancer stem cell-*like* phenotype, driving deregulation of core BCSC markers, such as CD24/CD44, activating EMT-associated transcriptional programs, and enhancing glycolytic metabolism, consistent with its well-established role in increasing breast tumor aggressiveness^23,43^. Within this context, the ALDH^high^ cell subset should be interpreted not as the sole driver of these phenotypic changes, but rather as an enriched and functionally amplified subpopulation that further enhances the stemness, metabolic flexibility, and invasive properties already initiated by POU1F1 overexpression. BCSCs display marked heterogeneity, spanning not only differences between tumor types but also diverse subpopulations that coexist within the same tumor^44^. In our model, the ALDH^high^ subpopulation is nested within the broader CD44^high^/CD24^low^ phenotype induced by POU1F1 across the entire cell population, and the ALDH^high^ subset emerges as an enriched and functionally reinforced population. The divergent behavior of MCF-7 and MDA-MB-231 cells further illustrates the context-dependent nature of plasticity. In MCF-7 cells, which have low endogenous POU1F1, forced expression activates multiple BCSC-related programs simultaneously. In contrast, MDA-MB-231 cells, which already possess high endogenous levels of POU1F1 and an intrinsic mesenchymal/CSC-like phenotype^45,46^, inhibition of POU1F1 weakly reduces some BCSC markers, such as CD44, but strongly reduces ALDH. This suggests that POU1F1 may function as a context-dependent regulator, promoting BCSC-*like* traits in luminal MCF-7 cells while maintaining them in triple-negative MDA-MB-231 cells.

Mechanistically, we described a connection between POU1F1, ALDH, and the IL-6/JAK2/STAT3 pathway. This pathway has been associated with pluripotency, therapy resistance, and metastasis^47^. After POU1F1 overexpression, there was a significant increase in the RNA-seq concerning the inflammatory response and the IL-6/JAK2/STAT3 pathway signature. RNA-seq data were confirmed by qPCR, showing increased IL-6 mRNA and protein levels in the culture medium, as well as other members of the IL-6 ligand family. The specific activation of STAT3 has been linked to the promotion of c-MYC, KLF4, and SOX9 expression, as occurs in our RNAseq after overexpression of POU1F1, which supports the self-renewal of BCSC-ALDH^high^, as well as mechanisms of chemoresistance^48,49^. Furthermore, STAT3 activation triggers downstream proteins such as AKT and ERK1/2, which are well-characterized for their roles in cellular metabolism, proliferation, metastasis, and CSC maintenance mechanisms^50^.

Janus kinase inhibitors and other drugs against the IL-6/JAK2/STAT3 pathway have been used to prevent the activation of the signaling pathway at various checkpoints, thus decreasing expression of stemness markers, i.e., CD44, CD133, ALDH, and EpCAM, and mammosphere formation in TNBC tumors^51^. Our data agree with these studies, demonstrating the functional effects of JKIs and IL-6R blockade on ALDH expression and colony and mammosphere formation, suggesting these drugs as a possible treatment in breast tumors with POU1F1 overexpression. Interestingly, in previous studies^23^, we have demonstrated POU1F1 expression in 89 of 110 invasive ductal human breast carcinomas, with strong expression in 12% of tumors.

In summary, our study shows that POU1F1 enhances breast cancer stem cell-like features and reveals its role in the IL-6/JAK2/STAT3 pathway and in ALDH regulation. Our results highlight the potential use of JAK inhibitors or anti-IL-6R antibodies to target BCSC-*like* cells in breast tumors with POU1F1 overexpression, by disrupting the IL-6/JAK2/STAT3/ALDH1A1 axis and thus preventing tumor recurrence.

## Methods

### Cell culture and drugs

The human breast adenocarcinoma MCF-7 and MDA-MB-231 cells were obtained from ATCC-LGC (Barcelona, Spain). Cell lines were grown in DMEM (Sigma, St Louis, USA) supplemented with 10% FBS, 100 U/ml penicillin-streptomycin (Thermo Fisher Scientific, Waltham, USA), at 37 °C in 5% CO_2_. The human mammary epithelial cell (HUMEC-TERT) was obtained from BIOCAT (Heidelberg, Germany) and grow in DMEM F12 supplemented with 5% horse serum, 0.5% penicillin/streptomycin, 0.1 mM glutamax, and 12.5 ng/ml ascorbic acid (Thermo Fisher Scientific), 0.5 µg/ml hydrocortisone and 1% fetal bovine pituitary extract (STEMCELL Technologies, Vancouver, Canada), 10 µg/ml insulin, 20 ng/ml hEGF, and 100 ng/ml cholera toxin (Thermo Fisher Scientific) at 37 °C in a 5% CO_2_. Cell lines were tested and authenticated by microscopic morphology, growth curve analysis, and mycoplasma detection according to the ECACC cell line verification test recommendations. The conditioned medium (CM) from MCF-7 cells overexpressing POU1F1 (CM-POU1F1) used to evaluate ALDH1A1 mRNA expression was obtained as follows: 5 × 10⁵ POU1F1 cells were cultured for 72 h in DMEM, the medium was centrifuged for 5 min at 300 × *g*, the supernatant was collected and used immediately. MCF-7 cells were treated with CM-POU1F1 for 72 h.

Tamoxifen was administered to cells at 10 μg/ml every other day and 10 mg/kg for 28 days in vivo. This study used two Janus kinase inhibitors, Ruxolotinib (a dose of 498 nM) and Ag490 (a dose of 15 nM) (STEMCELL Technologies), and an anti-IL-6 receptor (IL-6R) antibody, Tocilizumab (TCZ, a dose of 30 μg/ml, Thermo Fisher Scientific). IL-6 (Merck, Darmstadt, Germany) was used at a dose of 100 ng/ml. Treatments with Ruxolotinib, Ag490, TCZ, and IL-6 were performed for 72 h for qPCR and flow cytometry, for 7 days in colonies, and for 7 days in mammospheres.

### Plasmid and transfections

Stable overexpression of POU1F1 in the MCF-7 cells (POU1F1 cells) was achieved through lentiviral infection of a POU1F1 overexpression plasmid (pLV-puro-EF1A > hPOU1F1/FLAG) obtained from Vector Builder (Chicago, USA), which contains an ORF clone of the human POU1F1 gene. The empty vector (pLV-puro-EF1A) was used as a control. POU1F1 blockade was performed using a pool of 3 target-specific lentiviral vector plasmids, each encoding 19–25 nt (plus hairpin) shRNAs to knockdown the POU1F1 gene expression (Santacruz Biotechnology, Heidelberg, Germany). A pool of 3 scrambled shRNA sequences was used as a control.

### Cell viability, migration, and invasion assays

Cell viability/proliferation was measured using an MTT assay (Thermo Fisher Scientific). Cells were seeded in 6-well plates at a density of 5 × 10⁴ cells per well. After 72 h, absorbance was measured at 590 nm using an LB 940 Mithras multi-plate reader (Berthold Technologies, Shanghai, China).

Wound-healing assays were performed using an insert system in 35-well mm plates (IBIDI, Martinsried, Germany). Cells (3 × 10⁴) were seeded in each insert in DMEM. After 24 h, the inserts were removed, and the plates were placed under the Widefield Leica DMI 6000B microscope (Leica) to conduct a 12 h time-lapse analysis. Invasion assay was performed in 24-well plates containing inserts with 8-μm pores (Corning, New York, USA). At the top of the inserts, a three-dimensional structure resembling the extracellular matrix was created using Geltrex (Thermo Fisher Scientific), and 2.5 × 10⁴ cells were seeded in serum-free DMEM. Protein extract (200 μg/ml) of brain, liver, lung, and muscle from one 8-week-old female C57BL/6 mouse (obtained from Santiago de Compostela University animal facilities) was added to the bottom of the inserts. Protein extraction was performed after cutting small tissue fragments, placed in RIPA lysis buffer, and homogenized using a TissueLyser II (Qiagen, Hilden, Germany) with metallic beads for 2 min at 30 Hz. Protease inhibitors (Protease inhibitor cocktail, Sigma-Aldrich) were added to the lysis buffer to prevent protein degradation. After 24 h of incubation, cells that migrated through the pores onto the bottom of the insert were fixed in 90% cold methanol and stained with crystal violet (Sigma). The total number of invading cells was determined by counting the cells on the lower surface of the insert using an inverted microscope (Olympus IX73, Olympus, Allentown, USA).

### Colony and spheroid/mammosphere formation assays

Colony formation assays were performed in 6-well plates with 100 cells/well. Colonies were grown for 7 days, fixed with 90% methanol for 30 min at RT, and stained with crystal violet solution (Sigma) for 30 min. Colonies were counted directly on the plate. For three-dimensional (3D) mammosphere formation, 500 cells/well were seeded on low-attachment Nunclon Sphera plates (Thermo Fisher Scientific), maintained under standard culture conditions, and images acquired on days 1, 14, and 21. Mammosphere volume was calculated using the formula: π/6 × length × width^2^. Mammospheres were stained using a triple fluorescent staining protocol. Briefly, intact mammospheres were incubated for 1 h at 37 °C, protected from light, with Hoechst dye (1 μg/μl, Hoechst 33258, Invitrogen, ThermoFisher, Waltham, USA) to label all nuclei of live cells, caspase-3/7 (8 μg/μl, CellEvent Caspase 3/7 green detection reagent, Invitrogen) to identify apoptotic cells, and propidium iodide (PI, 1 μg/μl) to detect dead cells. Staining was performed on live, non-fixed mammospheres. Following incubation, mammospheres were gently washed to remove excess dye and immediately subjected to imaging. Mid-section optical planes were acquired using a confocal microscope (Cell Discoverer 7, Zeiss, Oberkochen, Germany) under identical laser intensity, gain, and exposure settings for all samples. Fluorescence intensity was quantified in three independent experiments by analyzing three mammospheres per condition. For live‑cell density, the 405 nm (blue) signal was measured by selecting a central region of interest (ROI) in each mammosphere using Fiji Image software, followed by background subtraction from a cell‑free area within the same image. Apoptotic and dead cells were analyzed using the same procedure, measuring fluorescence at 488 nm (green) and 568 nm (red), respectively. All measurements were expressed as relative units (RU).

### RNA isolation and qPCR

Total RNA for massive sequencing (RNA-seq) and qPCR was isolated using the commercial DNA, RNA, and protein purification kit (Macherey-Nagel, Düren, Germany). cDNA was synthesized with M-MLV-RT (Invitrogen, Thermo Fisher Scientific), and reactions of quantitative real-time PCR were done using SYBR Green PCR Master Mix (Thermo Fisher Scientific). The samples were denatured at 95 °C for 10 min, annealed at 55 °C for 15 s, and extended at 72 °C for 40 sec for a total of 40 cycles on Applied Biosystems™ QuantStudio™ qPCR Systems (Thermo Fisher Scientific). Primers targeted against the genes of interest are listed in Supplementary Table 1.

### Western blot, ELISA, immunofluorescence (IF), histology, and immunohistochemistry (IHC)

Proteins were extracted using a RIPA lysis buffer (Thermo Fischer Scientific) supplemented with protease and phosphatase inhibitors. Cell extracts (30 μg) were denatured at 95 °C, subjected to SDS-PAGE, and transferred to PVDF membranes (Merck Millipore, Burlington, USA). After blocking with Intercept Protein-Free Blocking buffer (LI-COR Biotechnology, Lincoln, USA), membranes were incubated overnight at 4 °C with anti-POU1F1 antibody (Sigma), anti-GPDH, and anti-β-actin (Santacruz), anti-JAK2, anti-pJAK2, anti-STAT3, anti-pSTAT3, anti-ERK1/2, anti-pERK1/2, anti-AKT, and anti-pAKT (Cell Signalling, Danvers, USA) antibodies for their expression and phosphorylation. A fluorescent secondary antibody was then applied, and detection was performed using the Li-Cor Odyssey fluorescence system at 700 and 800 nm wavelengths. An ELISA assay to determine IL-6 protein content released to culture medium was carried out using a human IL-6 ELISA kit as per the manufacturer’s instructions (Proteintech, Planegg-Martinsried, Germany). Antibodies are listed in Supplementary Table 2.

IF assays were performed on glass coverslips pretreated with poly-L-lysine for 1 h at RT. A total of 2 × 10⁴ cells were seeded on the coverslips, and 24 h later, cells were fixed in cold 96% ethanol, permeabilized with PBS + 0.3% Triton + 0.1 M glycine for 10 min at RT and blocked with 1% BSA in PBST for 30 min. Primary antibodies were incubated with 0.1% BSA in PBST overnight at 4 °C. After addition of the secondary antibody in 1% BSA, coverslips were mounted using DePex mounting medium Gurr (VWR, Barcelona, Spain) containing DAPI for nuclear staining. IF was analyzed using a Leica DM4B digital upright fluorescence microscope (Leica, Wetzlar, Germany).

For histology, samples were fixed in 10% neutral buffered formalin for 24 h and routinely embedded in paraffin. Section 4-μm**-**thick were cut with a microtome and stained using a standard H&E procedure. Sections were studied in an Olympus BX51 microscope (Tokyo, Japan) equipped with a DP70 digital camera (Olympus). Mitotic count was performed by counting the total number of mitoses per 10 high-power fields (HPF) (40x objective), as used to establish the Nottingham grade in breast cancer^52^.

For IHC, mouse tumor xenografts were immersion-fixed in 10% neutral buffered formalin for 24 h and routinely embedded in paraffin. Section 4-μm-thick were mounted on FLEX IHC slides (Dako-Agilent, Carpinteria, USA), deparaffinized, and subjected to heat-induced epitope retrieval in EnVision FLEX target retrieval solution (Agilent) for 20 min at 97 °C, using low-pH buffer for Ki-67 and high-pH buffer for CD44. Automated IHC was performed using the Autostainer Link 48 system (Dako-Agilent). Slides were incubated at room temperature for 20 min with primary antibodies: (1) anti-Ki67 (clone MIB1) mouse monoclonal antibody ready to use (Dako-Agilent) + Envision FLEX+ Mouse Linker (Dako-Agilent) for 15 min, and (2) anti-CD44 (clone DF1485) mouse monoclonal antibody at 1/25 (Dako-Agilent). Detection was carried out with EnVision FLEX/HRP reagent (a dextran polymer conjugated to horseradish peroxidase and goat anti-mouse immunoglobulins) for 20 min, followed by visualization with 3,3′-diaminobenzidine (DAB) chromogen solution for 10 min, and counterstaining with EnVision FLEX hematoxylin for 15 min. Slides were evaluated by an expert pathologist. To assess the proliferation index, Ki67 was quantified using a PathScan Combi scanner (Excilone, Elancourt, France). CD44 was graded in four categories, as proposed by ASCO/CAP in guidelines for HER2 testing: 0, no staining observed or membrane staining is faint/barely perceptible in ≤10% of tumor cells; 1+, incomplete membrane staining that is faint/barely perceptible in >10% of tumor cells; 2+, weak to moderate complete membrane staining observed in >10%) of tumor cells; and 3+, circumferential (complete) membrane staining that is intense/strong and observed in >10% of tumor cells.

### Flow cytometry, ALDEFLUOR assay, and cell sorting

Flow cytometry for CD24, CD44, and CD133 protein expression was carried out after labeling cells with the anti-CD24, anti-CD133, and anti-CD44 antibodies (BD) and analyzed using the Accuri Ruo Special Ryder System BD cytometer (BD) and/or the CytoFlex S cytometer (Beckman Coulter). To measure aldehyde dehydrogenase (ALDH) enzymatic activity, the ALDEFLUOR™ kit (STEMCELL Technologies) was used. Cell sorting was performed with the S3e Cell Sorter (BIO-RAD) to select POU1F1 cells with high ALDH activity (POU1F1-ALDH^high^). Data analyses were performed with FlowJo 10.0.

### Seahorse extracellular flux assays

Oxygen consumption rate (OCR) and extracellular acidification rate (ECAR) were measured using a Seahorse XF Flex Analyzer (Agilent Technologies, Santa Clara, USA). Cells (15 × 10³ per well) were seeded into XF96 microplates and cultured for 24 h. Prior to analysis, culture medium was replaced with Seahorse XF basal medium supplemented with 10 mM glucose, 1 mM pyruvate, and 2 mM glutamine (pH 7.4), and plates were incubated at 37 °C in a non-CO₂ incubator for 60 min. *Glycolytic function analysis:* Glycolytic activity was assessed using the Seahorse XF Glycolytic Rate Assay Kit (Agilent Technologies). ECAR was measured at baseline, followed by sequential injection of rotenone/antimycin A (0.5 µM) to inhibit mitochondrial respiration and 2-deoxyglucose (2-DG, 50 mM) to inhibit glycolysis. Basal glycolysis and compensatory glycolysis were calculated according to the manufacturer’s instructions. *Mitochondrial respiration analysis:* Mitochondrial function was assessed using the Seahorse XF Cell Mito Stress Test Kit (Agilent Technologies). OCR was recorded at baseline followed by the sequential injection of oligomycin (1.5 µM), carbonyl cyanide-4 (trifluoromethoxy) phenylhydrazone (FCCP, 1 µM), and rotenone/antimycin A (Rot/AA, 0.5 µM). Basal respiration, spare respiratory capacity, proton leak, ATP-linked respiration, and non-mitochondrial oxygen consumption were calculated according to the manufacturer’s instructions. OCR and ECAR values were normalized to protein content for accurate comparison between experimental conditions.

### Tumor-initiation analyses (limiting dilution experiments) and resistance to hormone- and radiotherapy

All animal studies were approved by the University of Santiago de Compostela Ethics Committee for Animal Experiments (code 15012/2020/013). For tumor-initiation capacity (TIC), 8-week-old female immunodeficient mice NRj-Foxn1^nu/nu^ (Janvier Labs, Le Genest St Isle, France) per group were used. Three types of cells were employed: control MCF7 (transfected with the empty vector), POU1F1 (MCF-7 cells with stable overexpression of POU1F1), and POU1F1-ALDH^high^ cells (with high ALDH expression after sorting POU1F1 cells). Subcutaneous tumors were generated in the fourth mammary gland by inoculating two cell dilutions (500 cells, *n* = 8 mice per group, and 5000 cells, *n* = 7 mice per group). Cells were previously transfected with a pCDNA3.1-Hyg-CMV-fireflyLuciferase vector to monitor tumor initiation and growth. After Luciferin injection (150 mg/kg), tumor growth was monitored by luminescence using the In Vivo Imaging System (IVIS, Caliper Life Sciences, Alameda, USA). An intensity map was obtained using the Living Image software (Caliper Life Sciences). The software uses a color-based scale to represent the intensity of each pixel (from blue representing low to red representing high). The TIC frequency was calculated using the Limiting Dilution Analysis software (https://bioinf.wehi.edu.au/software/elda/). Finally, tumors were removed at 8 weeks, and H&E staining was performed.

Female mice (age-matched, 8 weeks) with immunodeficiency Athymic Nude-Foxn1^nu^ (ENVIGO, Indianapolis, USA) were used for xenograft studies to evaluate the resistance to treatments. Orthotopic tumors were generated in mice by inoculation into the mammary fat pad of: (a) 1 × 10^6^ MCF7 cells (*n* = 8 mice), (b) 1 × 10^6^ POU1F1-overexpressing MCF-7 cells (POU1F1, *n* = 8 mice), and (c) 1 × 10^6^ ALDH^high^ cells sorted from POU1F1-overexpressing MCF-7 cells (POU1F1-ALDH^high^, *n* = 8 mice) in 0.2 ml of DMEM (without FBS) and Matrigel (50:50, BD Biosciences). Tumor growth was monitored externally using a digital caliper. On day 15, mice were treated with Tamoxifen (TMX, 10 mg/kg) for 6 days—until day 21—when tumors were surgically removed under isoflurane anesthesia (Alvira, Barcelona, Spain). Tumor volume was calculated using the formula: π/6 × length × width^2^. H&E staining of tumors was performed. Seven days later—day 28—the mice were irradiated with a single dose of 10 gray in the tumor area, and on day 33, TMX was again administered daily (10 mg/kg) until death or sacrifice to analyze overall survival with the Kaplan–Meier method. Mice were sacrificed when primary tumors reached a volume higher than 1500 mm^3^ or when mice lost at least 20% of their total body weight.

### Positron emission tomography/magnetic resonance imaging (PET/MRI)

PET/MRI studies using ^18^F-Fluoro-2-deoxy-2-D-glucose ([^18^F] FDG) were performed on day 54 after tumor cell injection in 2 mice per group to study tumor metabolism. All animals were fasted for 12 h before the radiotracer injection. Fused PET and MRI images were acquired using a Bruker BioSpec 3T PET/MRI scanner (bore diameter 17 cm) equipped with actively shielded gradients (450–900 mT/m). After an overnight fast, 6.30 ± 0.38 MBq of ^18^F-FDG was injected into the tail vein of each animal. PET/MRI static acquisitions consisting of 10 min PET scan and 16 min MRI scan were performed. All PET images were reconstructed using the MLEM (maximum likelihood expectation maximization) 0.5 mm algorithm with 18 iterations, including scatter, randoms, and decay correction. The image pixel size was 0.5 × 0.5 × 0.5 mm3, with a FOV of 90 × 90 × 150 mm³. For MRI, a pilot scan was acquired using the Bruker Localizer protocol, with an acquisition time of 32 s. Then, an axial study was conducted using a FISP (Fast Imaging with Steady-state free Precession) sequence to cover the whole body with an Echo Time (ET) = 2.8 s, Repetition Time (RT) = 5.7 s, Averages (NA) = 5, 30.2 × 30.2 × 50 mm FOV and a matrix size of 120 × 120 × 100. Finally, animals were returned to their cages with free access to food and water. PET/MRI images were analyzed using AMIDE software. Initially, MRI images were utilized to identify the tumor region. Once the tumor regions were identified, a qualitative assessment of the [^18^F] FDG PET images was performed to evaluate tumor metabolism by visually inspecting the intensity and distribution of radiotracer uptake. To ensure a fair and consistent qualitative assessment, the color scale was uniformly adjusted across all PET images, maintaining the same scaling parameters during visualization to detect regions with increased metabolic activity.

### RNA sequencing (RNA-seq) analysis

RNA-seq was performed by Novogene (Cambridge, UK) on the Illumina NovaSeq6000 platform. High-quality reads were aligned to the human reference genome (Ensembl GRCh38, version 108) using the STAR software (v2.7.6). Once aligned, the reads were assigned to their corresponding transcripts using the htseq-count program (v0.13.5) with the “intersection-nonempty” setting to ensure that each read was mapped to a single gene. Gene expression analysis was performed using DESeq2 (v1.32.0) in R (v4.2.2), and the data matrices were enriched with additional information using BiomaRT (v2.52.0) in R. This enrichment included details such as official gene names, chromosomal location, transcription direction, and other relevant attributes. Other RNAseq data used in this study were obtained from the NCBI Gene Expression Omnibus (GEO) database (https://www.ncbi.nlm.nih.gov/geo/). Enrichment analysis of transcriptional profiles was performed with the GSEA program (Gene Set Enrichment Analysis; v4.3.2). Transcriptional profiles were compared using different gene collections and parameters: weighted, enrichment statistics, and T-Test gene ranking metric. After the analysis, enrichment plots were obtained, including the NES (Normalized Enrichment Score) and FDR (False Discovery Rate) values.

### Statistical and bioinformatic analysis

Values are expressed as mean ± SEM. Means were compared using Student’s t-test or analysis of variance (ANOVA) with Tukey-Kramer multiple comparison tests. The Mann–Whitney U test was used for nonparametric data with 95% confidence intervals. Correlations between mRNA expression levels were calculated using Pearson’s correlation coefficient. *P*-values below 0.05 were considered statistically significant. Kaplan–Meier overall survival curves in mice were analyzed using the Log-rank (Mantel-Cox) test. Survival analyses were performed using the Kaplan–Meier Plotter online platform (KM Plotter; http://kmplot.com/analysis/) to evaluate how breast cancer patient clinical outcome is related to POU1F1/ALDH1A1 mRNA expression. For each gene, patients were stratified into high- and low-expression groups using the auto-selected (median) cutoff provided by the tool. Kaplan–Meier survival curves were generated for relapse-free survival (RFS), overall survival (OS), and distant metastasis-free survival (DMFS). Significance levels are represented as *P < 0.05, **P < 0.01, ***P < 0.001, and ****P < 0.0001 compared to the control. GraphPad Prism program was used for graph generation and statistical analyses.

## Supplementary information

Supplemantary information

## Data Availability

Raw and processed data for sequencing studies are available from NCBI’s Gene Expression Omnibus and are accessible through GEO Series accession numbers GSE287812 (MCF-7 cells), GSE287732 (HMEC), with and without POU1F1 overexpression (POU1F1) (RNA-seq). All other data will be available from the authors upon request.
