# Supplementary material for: POU1F1 induces cancer stem cell-like traits in breast cancer cells by IL-6/JAK2/STAT3 activation and enrichment of ALDH
Source: NPJ Breast Cancer. 2026 Mar 19;12:70. doi: 10.1038/s41523-026-00929-w (PMC13168259; doi:10.1038/s41523-026-00929-w)

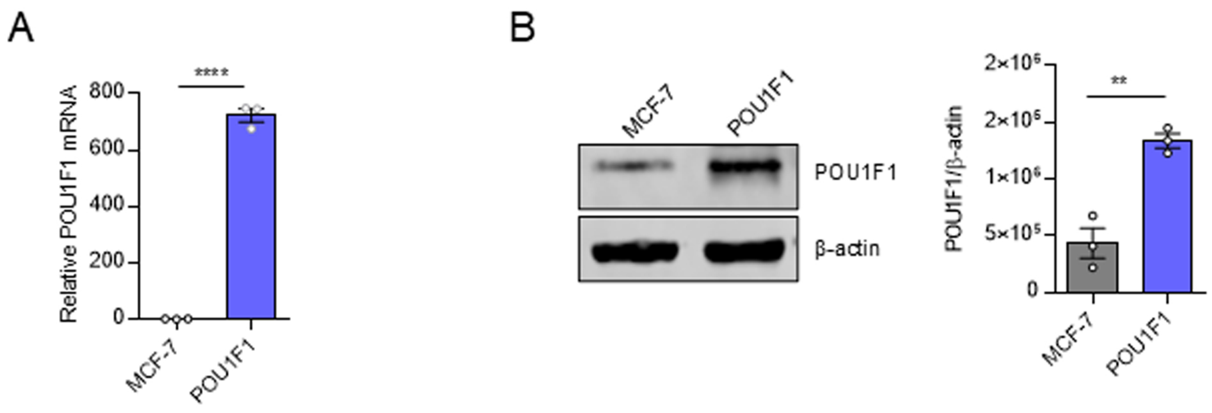

Supplementary Figure 1. Overexpression of POU1F1 in MCF-7 cells. A-B. POU1F1 mRNA and protein expression in control (MCF-7) and in POU1F1 stably transfected (POU1F1) MCF-7 cells.

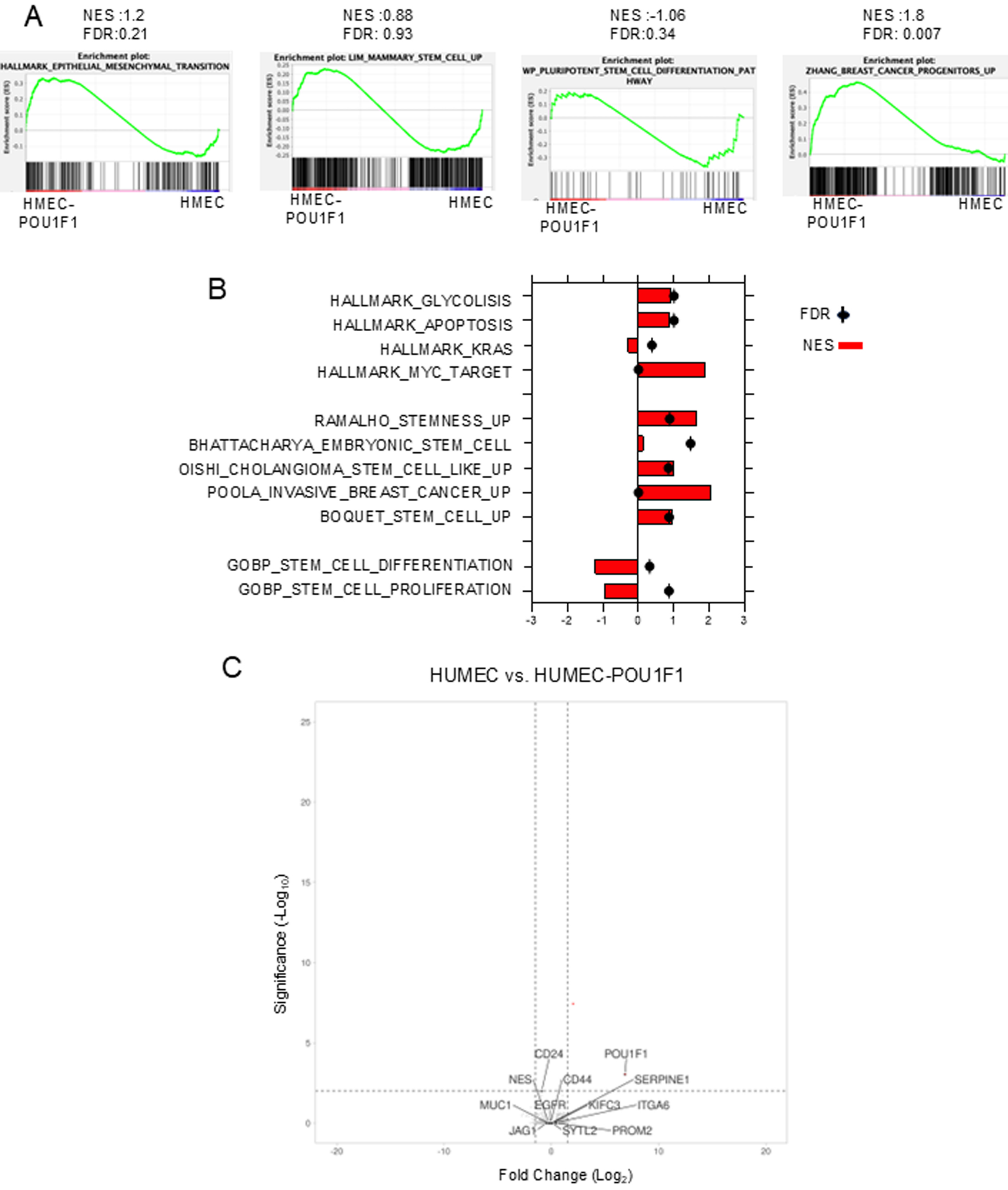

Supplementary Figure 2. RNA-seq data of HMEC cells without/with POU1F1 overexpression. A-B. GSEA plot of enrichment in EMT, mammary stem cell, pluripotent stem cell differentiation pathway, and breast cancer progenitors geneset and dataset enrichment graph of signature associated with stem cell data by RNA-seq from POU1F1-overexpressing HMEC-POU1F1 cells vs HMEC cells (GSE287732). **C.** Volcano plot in HUMEC vs. HUMEC-POU1F1 cells.

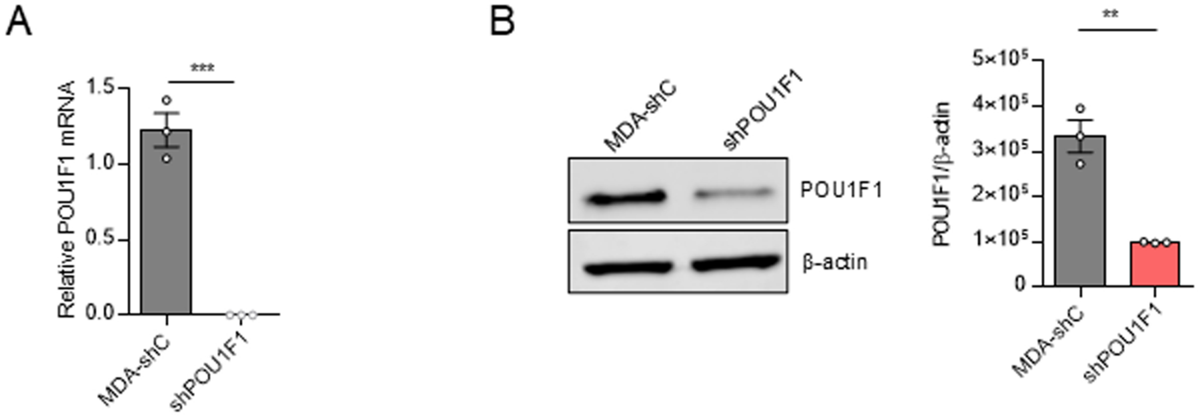

Supplementary Figure 3. Knockdown of POU1F1 in MDA-MB-231 cells. A-B. POU1F1 mRNA and protein expression in control MDA-MB-231 cells (MDA-shC) and after POU1F1 knockdown (shPOU1F1).

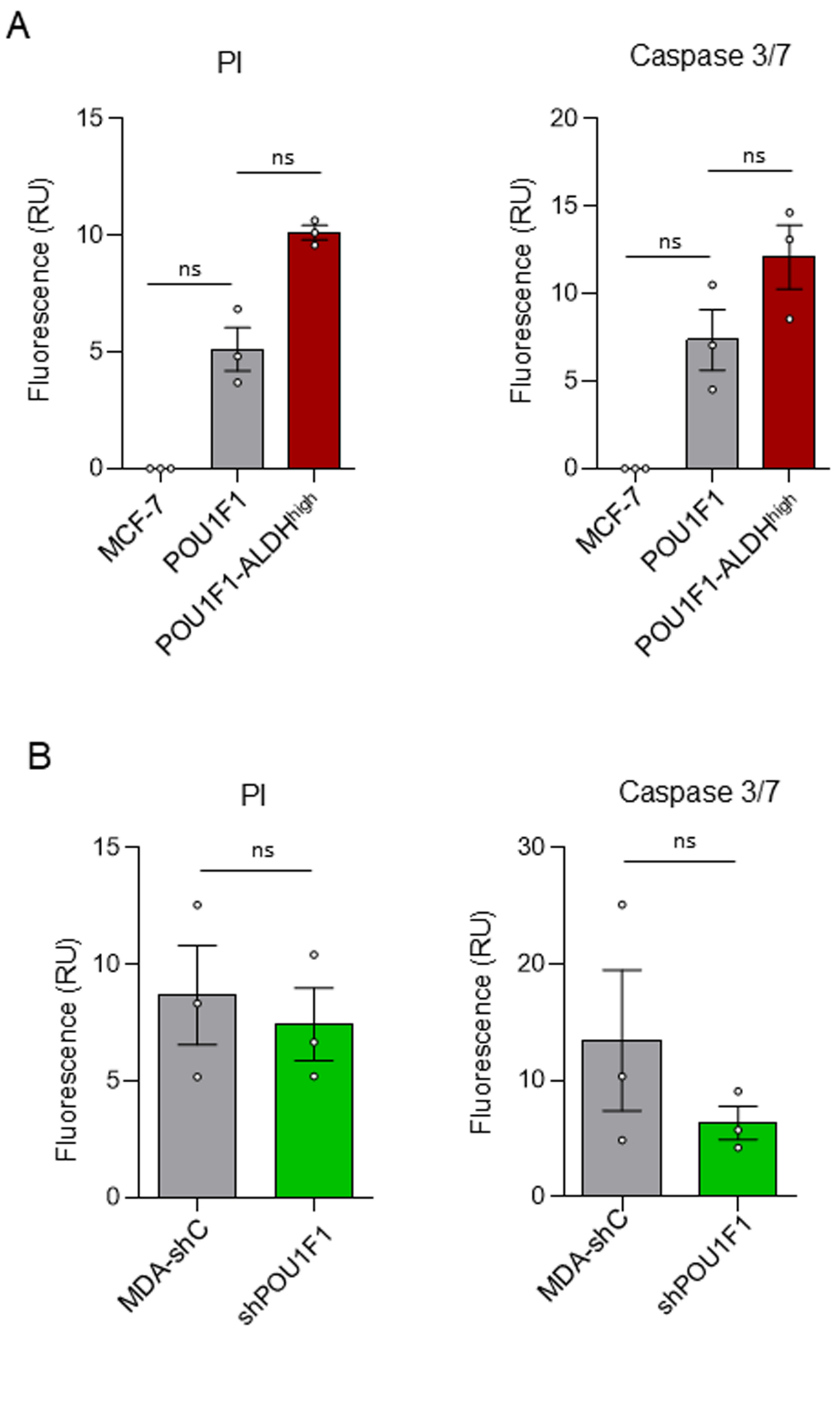

Supplementary Figure 4. Propidium iodide (PI, dead cells) and caspase 3/7 (apoptosis) fluorescence in mammospheres of (**A**) control cells (MCF-7), POU1F1, and POU1F1-ALDHhigh, and in (**B**) MDA-MB-231 control cells (MDA-sh) and with POU1F1 knock-down (shPOU1F1). ns= not significant. RU= relative units.

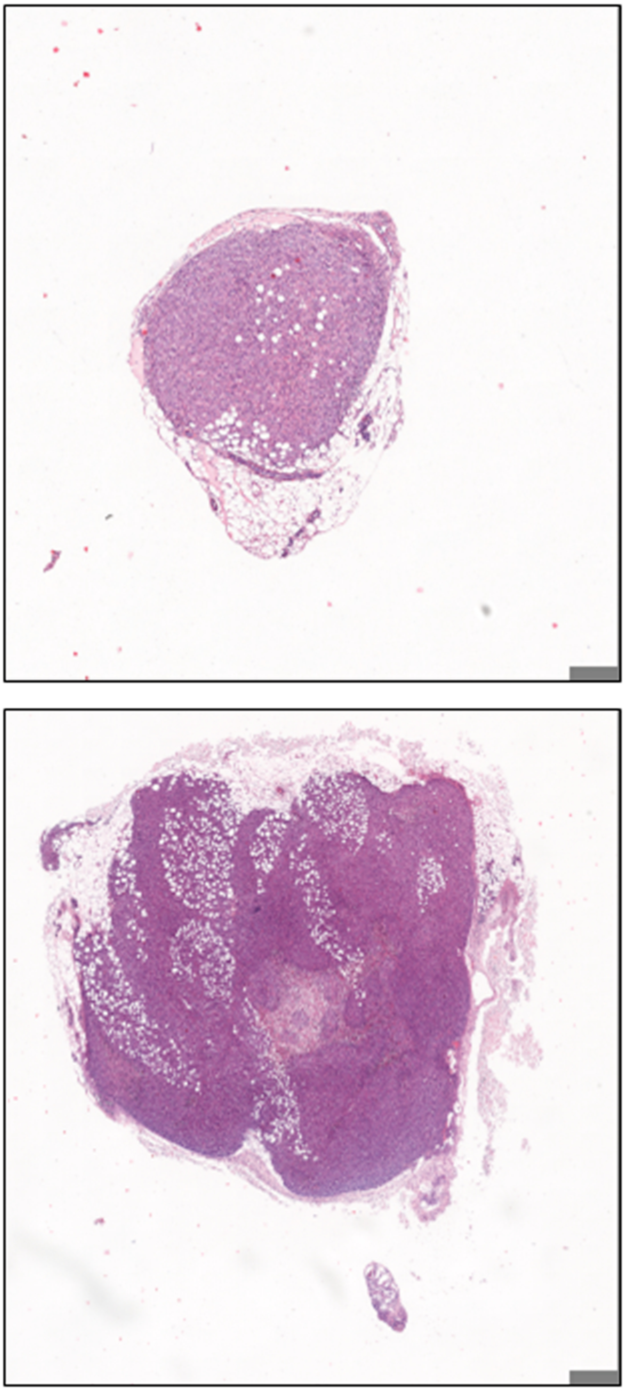

Supplementary Figure 5. Example of H&E of tumors from mice injected with MCF-7 cells (upper) and POU1F1 cells. Note that both are at the same scale.

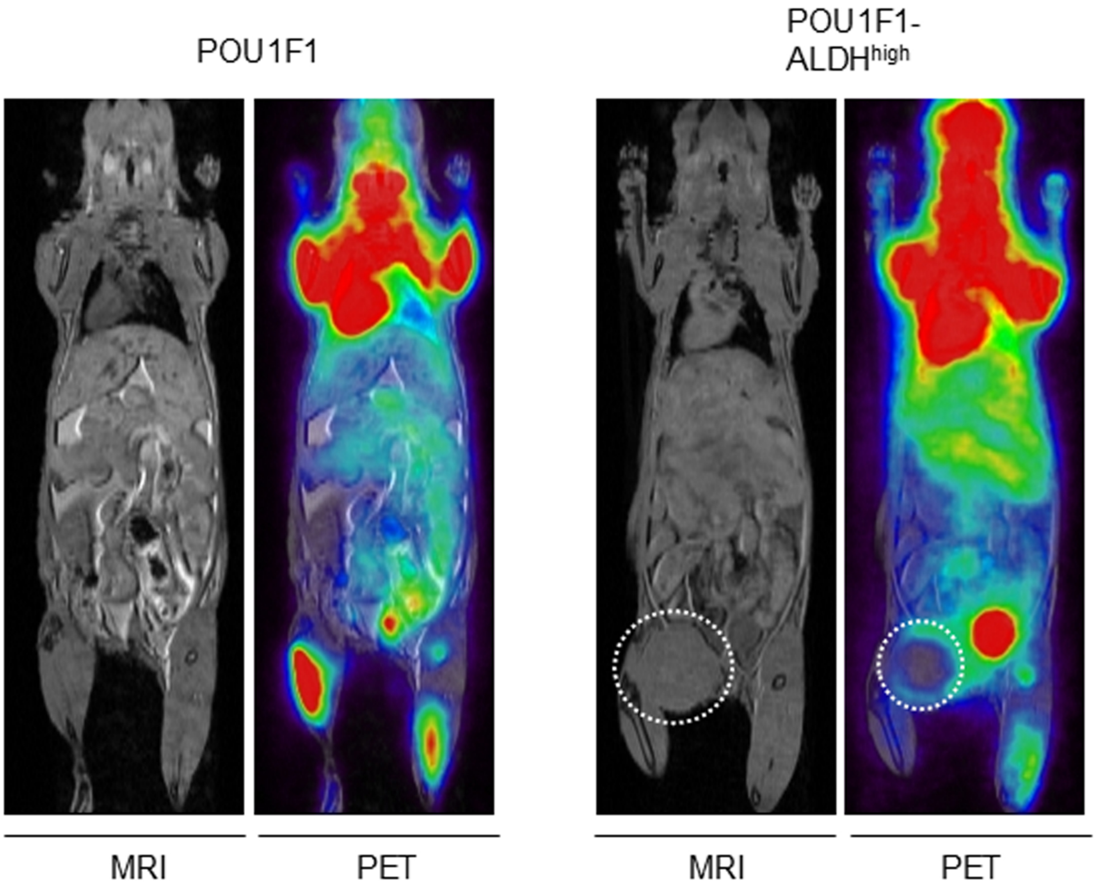

Supplementary Figure 6. [^{18}F] FDG PET/MRI assesses glucose uptake and tumor growth on day 54 in one mouse from POU1F1 and POU1F1-ALDH^{high} group. The dotted circle highlights the tumor in MRI/PET images from the POU1F1-ALDH^{high} group. In the POU1F1 group, no visible tumors are present.

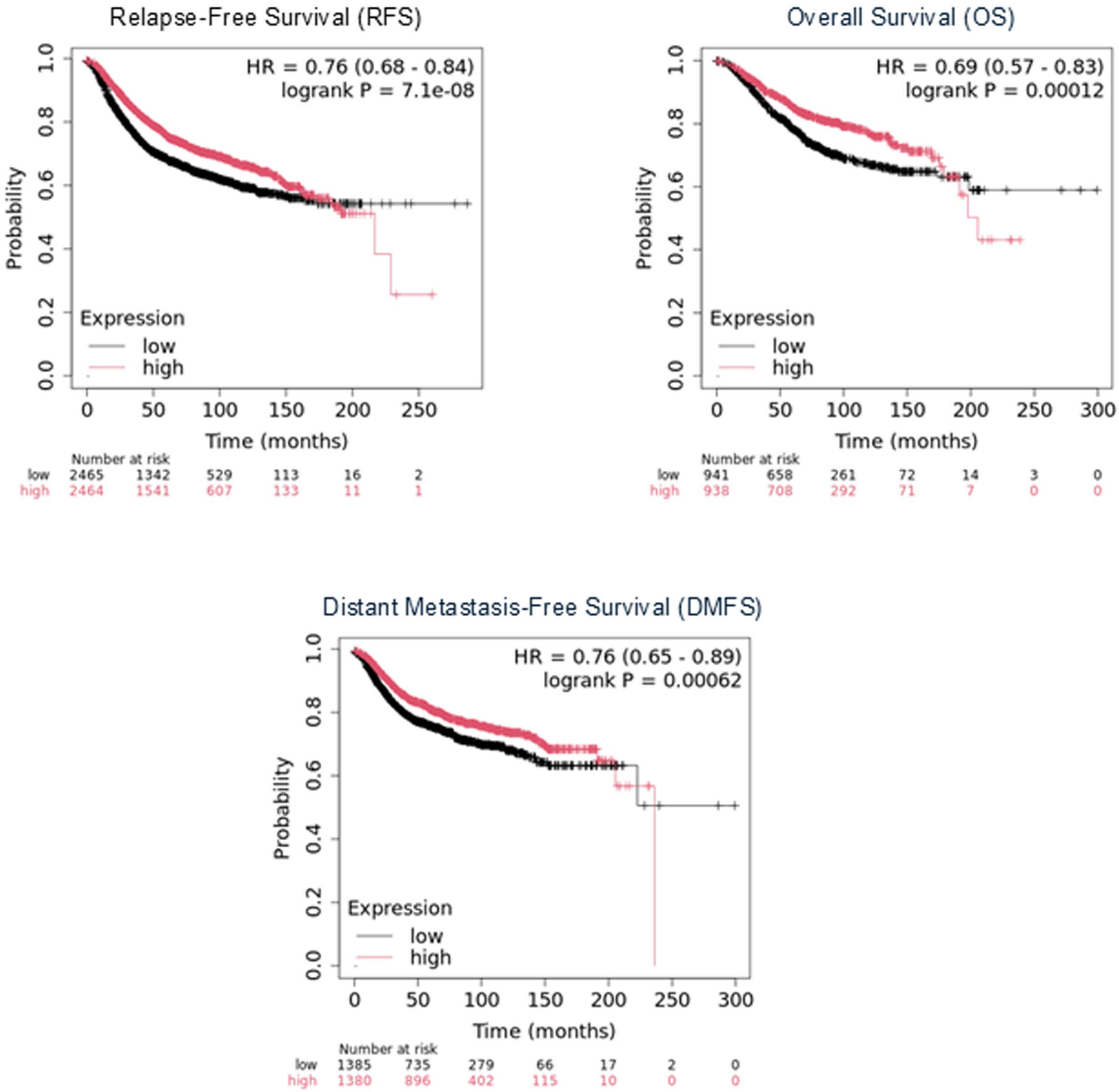

Supplementary Figure 7. Correlation between POU1F1 and ALDH1A1 mRNA expression and Relapse-Free Survival (RFS, n=4929), Overall Survival (OS, n=1879), and Distant Metastasis-Free Survival (DMFS, n=2765) in human breast tumors. Analyses were done using the KM plotter. Patients were stratified into high vs. low POU1F1 and ALDH1A1 mRNA expression using the median cutoff. Hazard ratios (HR) and log-rank *P*-values are shown in each panel.

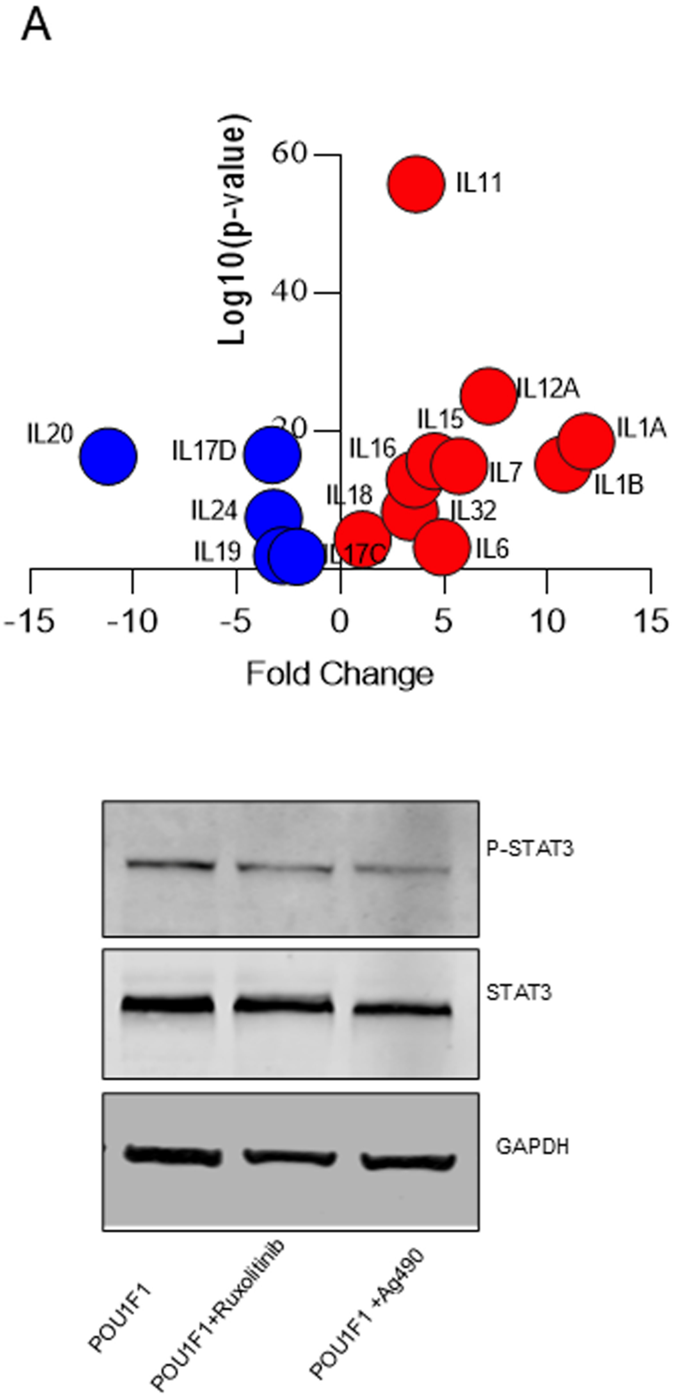

Supplementary Figure 8. **A**. Volcano plot showing downregulated (blue dots) and upregulated (red dots) interleukins mRNA in POU1F1 vs. MCF7 cells. **B**. Immunoblots of pSTAT3 and STAT3 after treatment of POU1F1 cells with the Janus kinase inhibitors Ruxolitinib (0.428 μM) and Ag490 (12 μM)

Supplementary Table 1. Primer sequences**.**

Primers	Sequences
Human *pou1f1* forward:	5'-TCCTGACCACACCTTGAGTC-3'
Human *pou1f1* reverse:	3'-CTTTTCCGCCTGAGTTCCTG-5'
Human *18s* forward:	5'-GTAACCGCTTGAACCCCATT-3'
Human *18s* reverse:	3'-CCATCCAATCGCTAGTAGCG-5'
Human *gapdh* forward:	5'-GTAACCCGTTGAACCCCATT-3'
Human *gapdh* reverse:	3'-CCATCCAATCGCTAGTAGCG-5'
Human *cd24* forward:	5'-ACCCACGCAGATTTATTTCCA-3'
Human *cd24* reverse:	3'-ACCACGAAGAGACTGGCTGT-5'
Human *cd44* forward:	5'-AAGGTGGAGCAAACACAACC-3'
Human *cd44* reverse:	3'-AGCTTTTTCTTCTGCCCACA-5'
Human *cd133* forward:	5'-TTCTATGCTGTGTCCTGGGG-3'
Human *cd133* reverse:	3'-GGGCCCATTTTCCTTCTGTC-5'
Human *epcam* forward:	5'-CTTTAAGGCCAAGCAGTGCA-3'
Human *epcam* reverse:	3'-CCAGTAGGTTCTCACTCGCT-5'
Human cd49f forward:	5'-AGAATTGACCTCCGCCAGAA-3'
Human cd49f reverse:	3'-TTTCAGCTTCAAGTGTGCCC-5'
Human *aldh1a1* forward:	5'-AAAGAAGCTGCCCGGGAAAAG-3'
Human *aldh1a1* reverse:	3'-CCCCATGGTGTGCAAATTCA-5'
Human *il-6* forward:	5'-CTTCAGGCCAAGTTCAGGAG-3'
Human *il-6* reverse:	3'-AGTGG ATCGTGGTCGTCTTC-5'

Supplementary Table 2. Antibodies used**.**

Antibodies	Used for	Source	Dilution
POU1F1	WB & IF	Sigma. Ref. 089K4863	1:5000
CD24	FC	BD. Ref. 555428	1:100
CD133	FC	BD. Ref. 566596	5 mg/ml
CD44	FC	BD. Ref. 555478	1:1000
GPDH	WB	Sc. Ref. 32233	1:5000
ß-actin	WB	Sc. Ref. 47778	1:5000
JAK2	WB	CS. Ref. D2E12	1:1000
p-JAK2	WB	CS. Ref. 3771	1:500
p-STAT3	WB	CS. Ref. 34911	1:400
STAT3	WB	CS. Ref. 9132	1:1000
p-ERK1/2	WB	CS. Ref. 9101	1:1000
ERK1/2	WB	CS. Ref. 9102	1:1000
p-AKT	WB	CS. Ref. 9271	1:1000
AKT	WB	CS. Ref. 9272	1:1000

WB: Western blot; IF: immunofluorescence; FC: Flow cytometry; Sc: Santa Cruz; CS: Cell Signaling.

WB POU1F1 in MCF-7 cells (Supplementary Fig. 1)

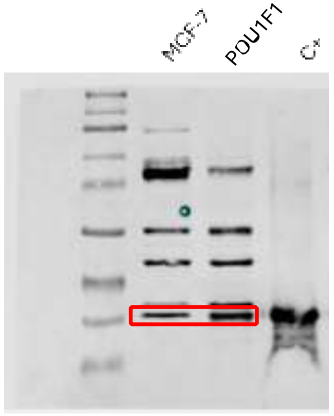

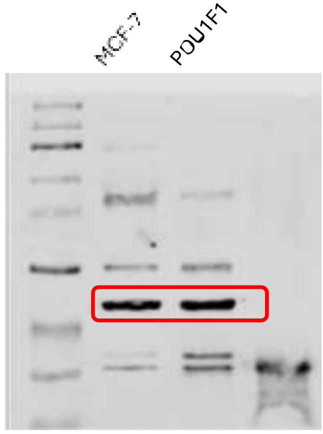

	WB1	WB2	WB3
**POU1F1**	MCF7-control-745000<br>MCF7- POU1F1-1450000	MCF7-control- 358000<br>MCF7- POU1F1-1230000	MCF7-control-373000<br>MCF7- POU1F1-1340000
**B-Actin**	MCF7-control-3360000<br>MCF7- POU1F1-3700000	MCF7-control- 2910000<br>MCF7- POU1F1-4770000	MCF7-control-2610000<br>MCF7- POU1F1-4570000

WB POU1F1 in MCF-7 cells (Supplementary Fig. 3)

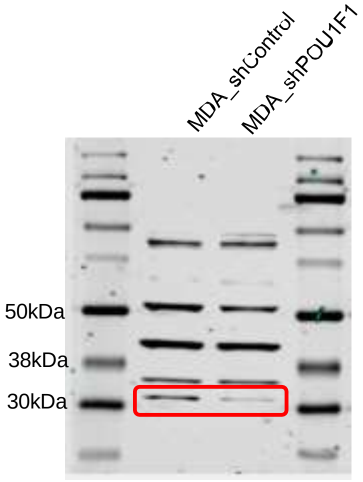

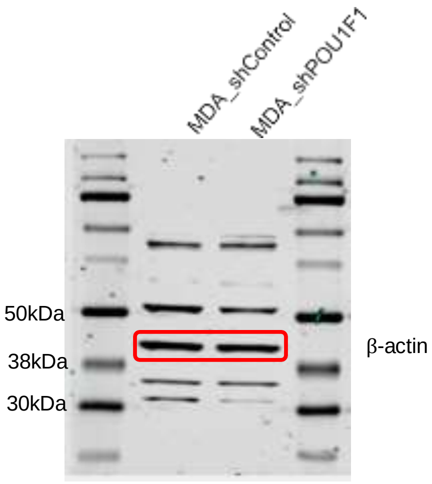

	WB1	WB2	WB3
**POU1F1**	MDA-shControl-116000<br>MDA-MB-231_sh POU1F1-272000	MDA-shControl- 118000<br>MDA-MB-231_sh POU1F1-395000	MDA-shControl-111000<br>MDA-MB-231_sh POU1F1-3340000
**B-Actin**	MDA-shControl- 1790000<br>MDA-MB-231_sh POU1F1-1490000	MDA-shControl- 1590000<br>MDA-MB-231_sh POU1F1-1890000	MDA-shControl-1450000<br>MDA-MB-231_sh POU1F1-1560000

WB pERK1/2 in MCF-7 cells (Fig. 6)

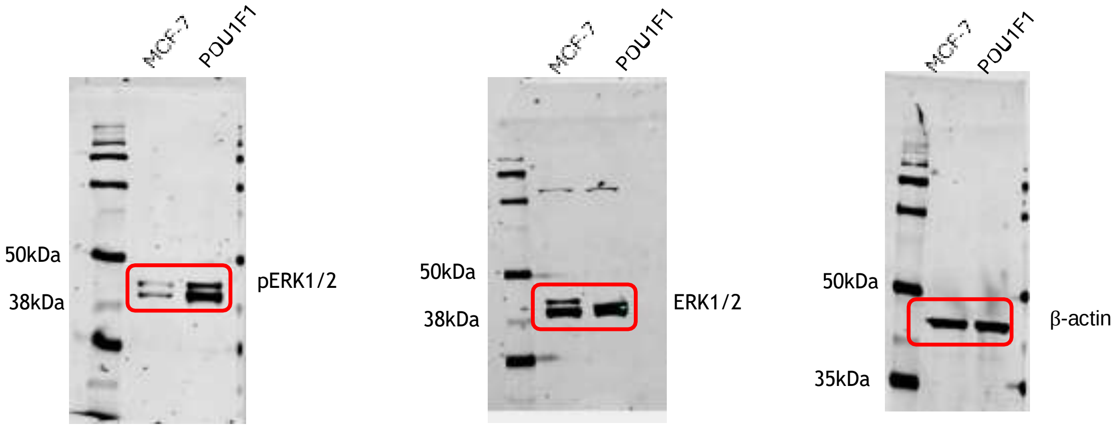

WB pSTAT3 in MCF-7 cells (Fig. 6)

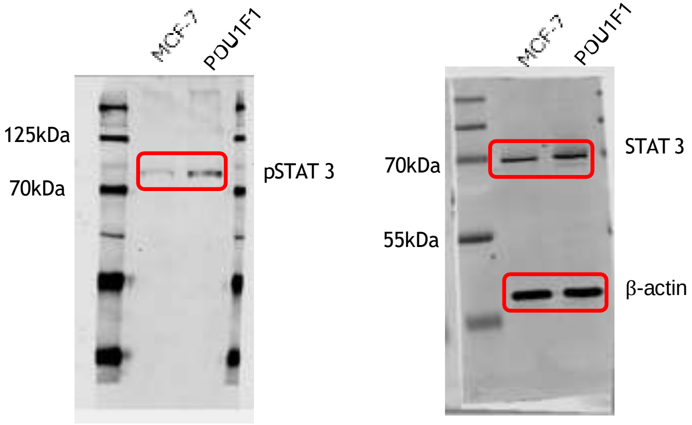

Wb pAKT in MCF-7 cells (Fig. 6)

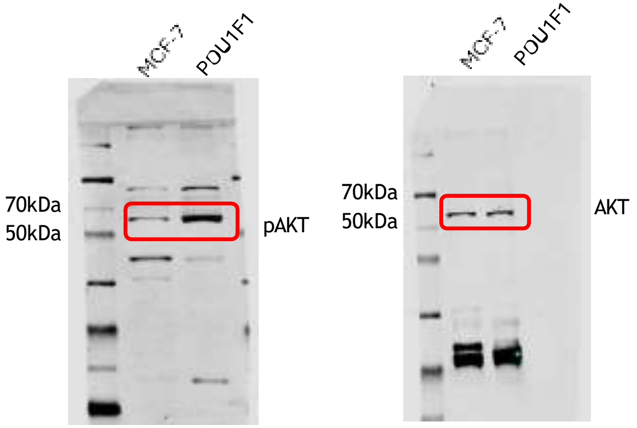

Wb Jak-2 in MCF-7 cells (Fig. 6)

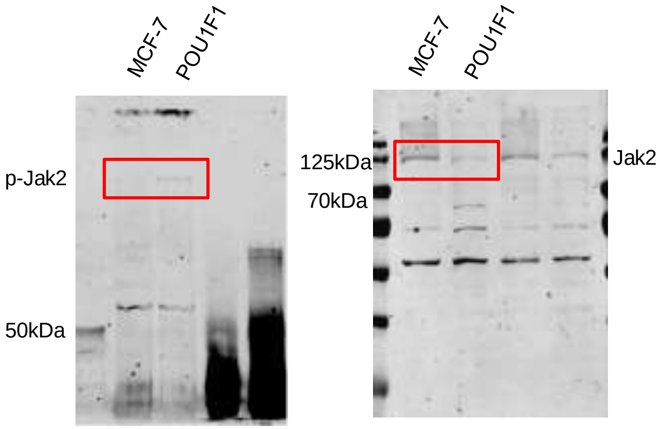

WB pSTAT3 (+/- Ruxolitinib and AG490 (Supplementary Fig. 6)

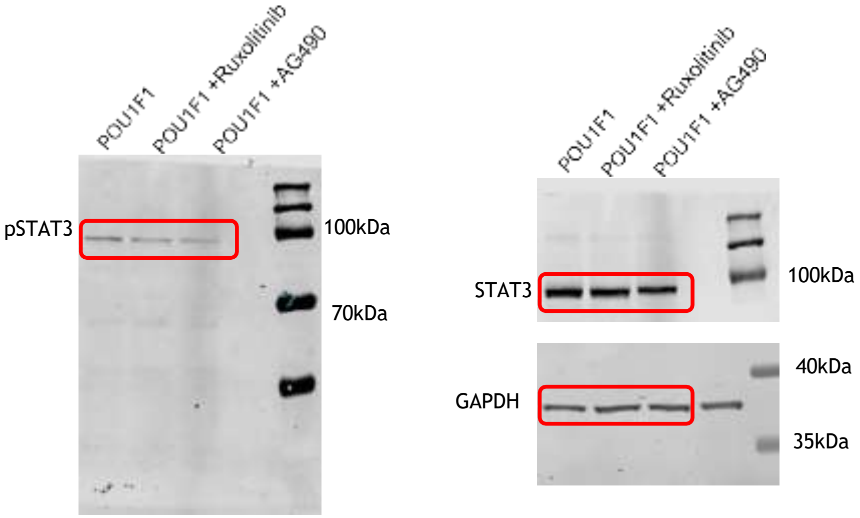

Supplement: Supplementary file 1 — Supplemantary information [file 41523_2026_929_MOESM1_ESM.pdf]
